# Attributes and generic competencies required of doctors: findings from a participatory concept mapping study

**DOI:** 10.1186/s12913-021-06519-9

**Published:** 2021-06-07

**Authors:** Kathryn Ogden, Sue Kilpatrick, Shandell Elmer, Kim Rooney

**Affiliations:** 1 Tasmanian School of Medicine, University of Tasmania, Locked bag 1377, Launceston, Tasmania 7250 Australia; 2 School of Education, University of Tasmania, Launceston, Tasmania Australia; 3 Centre for Global Health and Equity, Swinburne University of Technology, Hawthorn, Victoria Australia

**Keywords:** Medical education, Group concept mapping, Generic competencies, Attributes, Delivery of health services

## Abstract

**Background:**

Medical education should ensure graduates are equipped for practice in modern health-care systems. Practicing effectively in complex health-care systems requires contemporary attributes and competencies, complementing core clinical competencies. These need to be made overt and opportunities to develop and practice them provided. This study explicates these attributes and generic competencies using Group Concept Mapping, aiming to inform pre-vocational medical education curriculum development.

**Methods:**

Group Concept Mapping is a mixed methods consensus building methodology whereby ideas are generated using qualitative techniques, sorted and grouped using hierarchical cluster analysis, and rated to provide further quantitative confirmation of value. Health service providers from varied disciplines (including medicine, nursing, allied health), health profession educators, health managers, and service users contributed to the conceptual model’s development. They responded to the prompt *‘An attribute or non-clinical competency required of doctors for effective practice in modern health-care systems is...’* and grouped the synthesized responses according to similarity. Data were subjected to hierarchical cluster analysis. Junior doctors rated competencies according to importance to their practice and preparedness at graduation.

**Results:**

Sixty-seven contributors generated 338 responses which were synthesised into 60 statements. Hierarchical cluster analysis resulted in a conceptual map of seven clusters representing: value-led professionalism; attributes for self-awareness and reflective practice; cognitive capability; active engagement; communication to build and manage relationships; patient-centredness and advocacy; and systems awareness, thinking and contribution. Logic model transformation identified three overarching meta-competencies: leadership and systems thinking; learning and cognitive processes; and interpersonal capability. Ratings indicated that junior doctors believe system-related competencies are less important than other competencies, and they feel less prepared to carry them out.

**Conclusion:**

The domains that have been identified highlight the competencies necessary for effective practice for those who work within and use health-care systems. Three overarching domains relate to leadership in systems, learning, and interpersonal competencies. The model is a useful adjunct to broader competencies frameworks because of the focus on generic competencies that are crucial in modern complex adaptive health-care systems. Explicating these will allow future investigation into those that are currently well achieved, and those which are lacking, in differing contexts.

**Supplementary Information:**

The online version contains supplementary material available at 10.1186/s12913-021-06519-9.

## Background

The education of medical doctors has an important role to play in ensuring graduates are equipped for practice in modern health-care systems. In 1910 the Flexner Report promoted a university-based scientific model of medical education, leading to an education grounded in foundation scientific knowledge followed by clinical immersion [1]. One hundred years later, the anniversary of the Flexner Report prompted reflection on current medical education. On one hand Flexner has been lauded for the enormous contribution in bringing medical education into the twentieth Century progressive education movement [2], and on the other hand, arguments are made that the Flexner Report led to an individualistic, expert-centric culture which may now work against the collaboration needed in modern health-care [3]. The debate has led to discussion and speculation about what is required of medical education to produce doctors equipped to practice effectively over the next century [4–9].

Health and health-care have undergone an extraordinary transformation in the past 100 years in ways that Flexner could not have anticipated. Burgeoning knowledge and evidence-base about medical conditions and their management, coupled with a dramatic increase in preventable, non-communicable chronic illness and multi-morbidity, changes in community expectations of health-care, and increasing ethical and professional challenges have created a circumstance whereby the contemporary requirements of doctors continues to be re-evaluated [9].

Today’s doctors must have capabilities beyond core clinical knowledge and skills and medical education must embrace cultural change including generic capabilities such as working in collaborative teams, transformational leadership, innovation and improvement, and stewardship of funding [3]. In 2010 the global independent Commission on the Education of Health Professionals for the twenty-first century noted that “Health professionals have made huge contributions to health and socioeconomic development over the past century, but we cannot carry out 21st century health reforms with outdated or inadequate competencies” ([9] , p.1954). Effective teamwork and leadership are addressed to some extent only in some curricula, and talk of a patient-centred and team-based professional has failed to deliver on its promise [9]. Further, the importance of connection between education and health systems, whereby transformative professional education can lead to better health care systems, highlights a mismatch of competencies to patient and population priorities in current education systems [9].

While medical education prepares doctors with the knowledge, skills and attitudes to deliver high quality direct care to patients, the attributes and competencies required for newly trained doctors to understand or meet the requirements of delivery of care within a complex system are less explicit [10]. Required competencies for pre-vocational medical education are implicit in statements from regulatory bodies (for example: [11, 12]), and outlined more explicitly in frameworks that are largely driven by post-graduate education bodies (for example: [13–19]). However, the attributes that are required of doctors to achieve competencies are more obscure and guidance about how to achieve them in medical education is less clear. Enabling their development requires making these competencies overt and guiding the provision of learning opportunities [20–22]. These transformations necessitate reflection on the pedagogical strategies required to produce doctors for modern health-care systems [23–25]. Instructional design should be competency-led, with the requisite competencies identified and curriculum tailored accordingly [9].

In Australia, medical education occurs as either a 5-year undergraduate or 4-year post-graduate degree with a compulsory year of supervised practice (internship) prior to full registration with the regulatory body. The degree is generic, and specialisation occurs after graduation and internship. The regulatory body has since 2017 conducted regular intern readiness surveys with around 20% response rate. The survey results indicate that there are some areas for which interns feel more prepared, largely relating to core clinical skills and access to knowledge. They feel less prepared for providing nutritional care, error reporting, and certain aspects of familiarity with hospital systems and self-management skills [26]. A longitudinal study at an individual institution identified relative lack of preparedness in attributes such as the use of informatics, audit, self-management of own health, governance, dealing with error, and self-critique [27].

Clarifying and providing behavioural anchors to the attributes and competencies required to complement fundamental clinical knowledge and skills and enable doctors to be effective in modern health care systems, is essential for medical education to achieve its aims. Terminologies used to represent these attributes and competencies are varied, including ‘soft-skills,’ [28] ‘non-technical skills’ [29], ‘non-academic attributes’ [30], ‘non-cognitive attributes’ [31], ‘generic skills’ [32] and ‘personal attributes’ [33]. Collectively they can be considered as the scaffolding which enables doctors to work effectively within modern health-care systems to optimise the delivery of health-care. They are referred to here as attributes and generic competencies, where generic competencies are those which are not specifically clinical, albeit often carried out in a clinical setting. This study aims to develop consensus and make explicit these attributes and generic competencies. In recognizing that these are addressed in existing competency frameworks referred to above, we aim to elaborate by asking stakeholders what attributes and behaviours can lead to these competencies.

## Methods

### Overview

We used participatory Group Concept Mapping [34] to conceptualise the attributes and generic competencies required for effective practice in modern health-care systems. Logic model transformation and ratings of importance to practice and preparedness, sought from junior doctors, further develop the conceptualisation in the context of medical education.

Group Concept Mapping (GCM) provides a structured approach for consensus building, using quantitative and qualitative methods, allowing for the integration of input from multiple sources into a visual representation of a conceptual framework, and is described in detail by Kane and Trochim [34]. GCM leads to a visual representation of composite thinking of participants and stakeholder groups with the ability to engage in and represent complexity. An online platform supports the collection, management and analysis of data [35]. Stakeholders are engaged to generate ideas, sort the ideas into groups, and rate ideas according to value. The statistical techniques of multi-dimensional scaling and hierarchical cluster analysis aggregate data to reveal patterns through visualisation, allowing for interpretation to support further utility of the model. GCM is a structured applied social research methodology, to connect theory to observation and research to practice. It has been widely used in the health-care sector for policy and planning for health services [36–40] and increasingly in the medical education sector to understand educational processes and outcomes [41–43].

The GCM process consisted of four key phases: 1) idea generation, review and synthesis [34]; 2) sorting and rating facilitated by the online platform [35]; 3) analysis of data using quantitative and qualitative techniques to produce a visual concept map; and 4) confirmation and further interpretation of results using logic model transformation [44]. This study was approved by the Tasmanian Health and Medical Human Research Ethics Committee  (reference number H0015769).

### Participants

Two participant groups were engaged: 1) contributors to the concept map, and 2) respondents to the rating activity. Contributors were identified using a purposive sampling strategy which aimed to ensure broad representation from the following groups of people who are engaged in some manner in the health care system: patients and carers; clinicians from a array of disciplines; health-care managers; tertiary sector educators; and professional association representatives. We aimed to include a diverse contributing group who could comment from experience and differing perspectives, seeking face validity of items and that a full range of competencies were identified. Potential contributors were invited to participate in one or more stages of the project. Junior doctor respondents were recruited for the rating component. Approaches were made directly by researchers to generate and sort ideas, junior doctors were invited to participate via email by management at two hospitals in Tasmania, Australia. This study was conducted between October 2017 and October 2019 with participants across five of eight Australian states and territories.

### Generating ideas

Contributors to the concept map were invited via email to provide responses using an online platform [35]. They were asked to complete the focus statement '*An attribute or non-clinical competency required of doctors for effective practice in modern health-care systems is*...' as many times as they liked. Contributors were provided with the following definitions:

- Attribute: A quality or feature regarded as a characteristic or inherent part of someone or something and does not depend on acquired knowledge; and
- Non-Clinical Competency: Transferable, generic professional skills which are not rooted in the medical profession. They may be carried out in a clinical or non-clinical environment by health-care workers but are not uniquely clinical in nature (e.g. communication related skills).

Statements were iteratively reviewed, refined, and synthesised with duplicates and irrelevant ideas removed, and similar ideas combined. Guidelines for this review process included determining whether statements needed to be split into more than one idea, elimination of repeated ideas, elimination of statements which were not relevant to the focus statements (e.g. health-care specific clinical skills), and clarification of content if required to ensure ideas were concise and understandable [34]. We determined data saturation through iterative synthesis and comparison of ideas as they were generated onto the online platform. Once we were satisfied that the point of saturation had been reached a research advisory group was convened comprising five clinicians from the disciplines of nursing, medicine and psychology, and one consumer. The group members reviewed the statement list and provided feedback with regards to relevance of the statements to the research, clarity of statements, and completeness of the statement list to confirm saturation. A final set of statements detailing attributes and non-clinical competencies was generated.

### Sorting of statements

Contributors were invited to sort the statements into groups in a way that made sense to them [34] and provide a relevant name for each group. This activity occurred online using the Concept Systems Global Max^tm^ platform [35]. We set a minimum target of 30 sorters with representation from all stakeholder groups, which is in line with the recommended number (20–30) to provide reliable results while acknowledging that larger number of sorters yields higher inter-rater reliability estimates [45].

### Data analysis for cluster map

A cluster map was built and labels determined using the online Concept Systems Global Max analysis program [34, 35] which integrates qualitative and quantitative methods [46, 47], in addition to a qualitative sense-making process carried out by researchers and the advisory group.

A similarity matrix was created to identify how often statements were sorted together. Through the process of multidimensional scaling [48], this similarity matrix was then used to create a two-dimensional ‘point map’ of each statement to visually represent the sorting data, with statements sorted together more often placed closer on the map. A stress value statistic was generated, which indicates how well the 2-dimensional point map represents the raw sorting data [45] which is multi-dimensional.

Hierarchical agglomerative cluster analysis using Ward’s algorithm [49] was used to group statements into clusters. A bridging value was identified for each statement, indicating whether it was anchoring - sorted primarily with others close by, or bridging – sorted with others across a larger area of the map. The option of imposing a filter on the analysis which would require statements to be sorted together more than one time was explored but did not significantly change the outcome and therefore was not utilised.

Determining the number of clusters relied on qualitative review by researchers [47] using interpretive analysis [50]. Statements in each cluster were examined from maps with five through to 15 clusters, and using expertise in medical education and clinical medicine, the optimal cluster solution was determined [45]. This process was undertaken by one author (KO) and reviewed and confirmed by all other authors and the research advisory group. Examination of statements was then made to determine whether there were any statements placed on a cluster boundary which were deemed to better fit in an adjacent cluster and if so the boundary was changed.

Cluster labels were determined using three sources of information: GCM software provided a list of the 10 best fit labels provided by contributors [51]; the statement bridging values provided information about which statements are the most central to the cluster; and researchers read and synthesised their understanding of the statements in each cluster.

One author (KO) proposed cluster names, the other authors and research advisory group reviewed the decision and made alternative suggestions until agreement was reached. All contributors were provided with a provisional set of results and invited to make comment over a 2-week period. A further seven clinicians were interviewed and their feedback on the relevance and utility of the model sought (not reported here). Feedback was considered by the research team for incorporation into the models.

### Data analysis for logic model

Subsequently we developed a logic model as a tool to further operationalise the data incorporating inputs, processes and activities, and outputs [52, 53]. Impacts and outcomes are not incorporated in the model as they were not included as part of the initial concept mapping process, rather the logic model focuses on strategies [53]. Each statement was examined to determine whether it related to input, process or activity, or output elements. Statements which incorporated more than one of these categories were split into individual elements and re-worded to ensure that they were understandable. Each element was then grouped according to thematic similarity, starting with elements within the same cluster but incorporating those from other clusters if appropriate. Groupings were then examined for causal linkages between inputs, processes and activities, and outputs, including feedback loops. This process was performed by one author (KO) and the logic model reviewed by all other authors and the research advisory committee to provide input and ultimately confirm the model.

### Ratings

Junior doctors were invited to rate each of the statements generated in the above process using Likert scales according to the following two prompts:

1. Relatively how important is this attribute or competency to your role as a doctor? (1 = relatively less important to 5 = relatively more important)
2. How well prepared were you when you graduated? (1 = not prepared; 2 = somewhat prepared; 3 = reasonably prepared; 4 = well prepared; 5 = very well prepared)

**Fig. 1:**
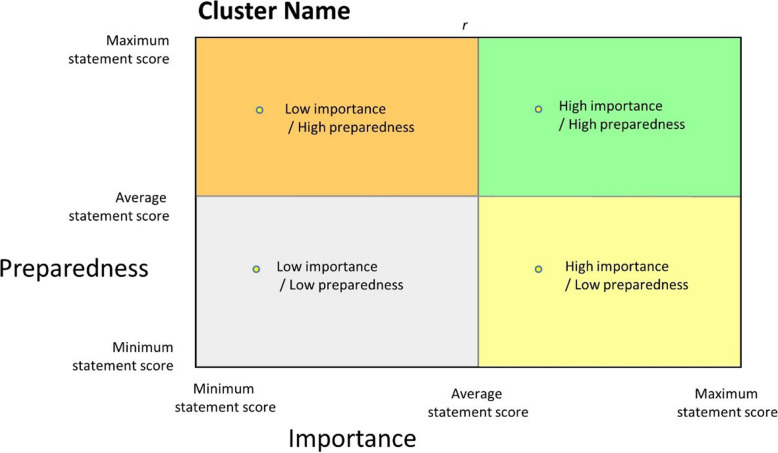
Go-zone template

Data were entered directly onto a web-based platform. Ratings for each statement were averaged, to provide indicative representation of the relative importance and preparedness as reported by respondents for each statement. The nature of the scale and significantly skewed data warrants caution in further analyses, however these averages were used to produce visual tools to enable a ‘birds-eye’ view of the data. Importance and Preparedness ratings were graphed against each other for all data and for each cluster to produce ‘go-zones’. Go-zones are a visual tool that allows for statements to be categorised, using their average rating, into one of four quadrants: high importance/high preparedness; high importance/low preparedness; low importance/high preparedness; and low importance/low preparedness. The high importance/low preparedness quadrant of particular interest (Fig. 1).

Averages were calculated for illustrative purposes for each cluster and clusters ranked according to least to most important, and least to most prepared. A visual ‘pattern match’ was produced which demonstrates for each cluster, relative importance and preparedness, allowing the easy identification of clusters which are perceived as more or less important, and how this relates to perception of preparedness.

## Results

### Participants

There were 67 contributors, 43 (62.7%) females, from across the stakeholder groups contributing to brainstorming (51) and to structuring the statements through sorting (37), they nominated up to two roles in health-care (Table 1). Most contributors were from the Australian state of Tasmania, with 10 from four other states across Australia. Thirty-seven hospital doctors responded to the rating component of the project. Of those who provided their sex (*n* = 35), 21 (60.0%) were female, 13 (37.1%) were male, and 1 (2.9%) identified as other. 24 (64.9%) were in the first three years post-graduation and 13 (35.1%) had been graduated > 3 years. Due to third party recruitment it is unknown how many people received an invitation to participate.

**Table 1:** Contributor roles in health-care (combined nominated primary and secondary roles, brainstorming and sorting only)

Role in health-care			Total
Consumers			19
	Patient	18	
	Carer	1	
Medical Practitioners			28
	General Practitioner	12	
	Physician	5	
	Anaesthetist	4	
	Surgeon	3	
	Prevocational doctor	2	
	Psychiatrist	1	
	Paediatrician	1	
Other Clinicians			17
	Nurse	8	
	Allied health practitioner	7	
	Psychologist	1	
	Pharmacist	1	
Academic			31
	Educator	22	
	Researcher	9	
Management and administration			11
	Health-care policy and administration	7	
	Practice management and administration	2	
	Community health organisation	2	
Pastoral carer			2
Industry organisation representatives			2
Health informatician			1
Coaching and performance specialist			1

### Attribute and generic competency clusters

Three hundred thirty-eight ideas were contributed which were iteratively reviewed and synthesised into statements, while at the same time evidence of saturation was sought. A detailed representation of this process is provided (Additional file 1, Item 1). The final statement list consisted of 60 attributes and generic competencies. Sorting data provided from 36 contributors led to a similarity matrix (Data – sorting similarity matrix provided). These were analysed and statements located in a two-dimensional point map with a stress value of 0.259, which, for this type of research, indicates a good fit between the raw sorting data and the two-dimensional representation of that data [45]. Contributor and advisory group review of the concept model led to minor changes in wording. Interviews were conducted to elaborate on its relevance and utility however are beyond the scope of this report.

**Fig. 2:**
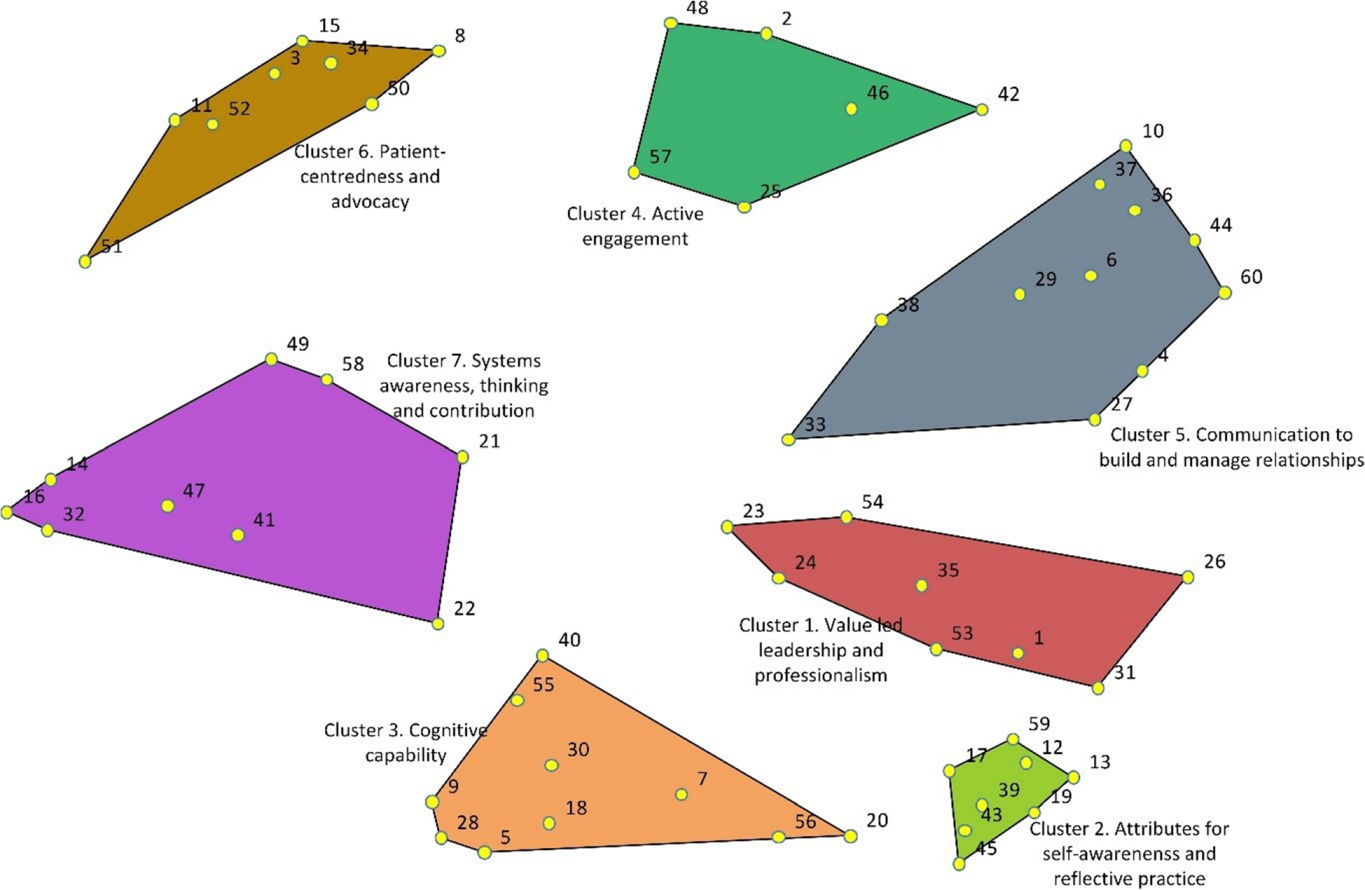
Seven Cluster Concept Map of Attributes and Generic Competencies for Effective Practice in Modern Health-care Systems. Sixty statements are represented by yellow dots and statement numbers. Statement details are available in Additional file 2

Hierarchical cluster analysis and interpretive analysis led to a seven-cluster concept map of attributes and generic competencies required of doctors for practice in modern health-care systems (Fig. 2). Yellow dots in Fig. 2 represent each statement and their number. Close examination of the statements within each cluster led to four statements being moved from one cluster to an adjacent cluster (Additional file 1, Item 2). A summary of the construct of each cluster is provided (Table 2), however the full list is fundamental to the interpretation and meaning of the overarching map (Additional file 2).

**Table 2:** Description of the elements and constructs within each cluster^a^

Cluster 1. Value-led professionalism	Cluster 1 is underpinned by a professional commitment and work ethic, integrity, empathy, initiative and willingness to make time when needed. Elements relate to effective role-modelling and leadership; and conduct in a manner that is consistent with community expectations.
Cluster 2. Attributes for self-awareness and reflective practice	Cluster 2 is underpinned by curiosity, self-awareness, insight, resilience and perseverance. The ability to reflect and learn from failures, an awareness of limitations, and ensuring own well-being are also highlighted.
Cluster 3. Cognitive capability	Cluster 3 is underpinned by attributes which lead to cognitive ability, including flexibility, analytical capacity, creativeness and innovation, situational awareness, resourcefulness and self-directed learning. Highlighted is the ability for decisive action, clarity of thought processes, and ability to manage uncertainty and ambiguity.
Cluster 4. Active engagement	Cluster 4 relates to a set of attributes and skills which promote full engagement between doctors and those who they work with – patients and colleagues. It includes the embracing of cultural diversity, responsiveness to the communication needs of patients, engaging in narrative, and ensuring seamless transfer of care through the health system.
Cluster 5. Communication to build and manage relationships	Cluster 5 is underpinned by exemplary communication skills including building rapport, demonstrating respect, active listening, open communication channels, and the effective use of written and modern communication technologies. Highlighted are skills in negotiation and conflict resolution, effective interpersonal dynamics and working relationships, trust, and ability to manage differing agendas.
Cluster 6. Patient-centredness and advocacy	Cluster 6 is underpinned by an approach to care which recognises the context in which patients exist, the importance of their priorities for care, and a willingness to advocate and prioritise activities for the benefit of patients. It is exemplified by an agile and pragmatic approach to the delivery of individualised care, ability to assist patients to navigate the health-care system, maintaining respectful relationships, and a commitment to the notion of co-creation of health
Cluster 7. Systems awareness, thinking and contribution	Cluster 7 is underpinned by an awareness and understanding of systems and the organisational aspects of health-care, an understanding of the doctor’s role within the system and the local community, leading collaborative care, commitment to the team, and courage to advocate for systemic change.

^a^See Additional file 2 for the full set of statements which have led to this summary

### Logic model transformation of statements

The 60 statements represented in the concept map were transformed into 51 input elements, 37 process/activity elements, and 35 output elements. This organisation of the data distinguished between attributes (input elements) and competencies (processes and activities), with the interaction of these leading to desirable outputs. Connections between elements were identified, including feedback loops, to produce a logic model. Through this process it emerged that there were three overarching domains or meta-competencies to the conceptual model, with interaction between the items from each cluster within the domains. These were:

1. *Leadership and systems thinking:* Incorporating Cluster 1: Value led professionalism and leadership, and Cluster 7: Systems awareness, thinking and contribution
2. *Learning and cognitive processes:* Incorporating Cluster 2: Attributes for self-awareness and reflective practice, and Cluster 3: Cognitive capability.
3. *Interpersonal capability:* Incorporating Cluster 4: Active engagement, Cluster 5: Communication to build and manage relationships, and Cluster 6: Patient-centredness and advocacy.

**Fig. 3:**
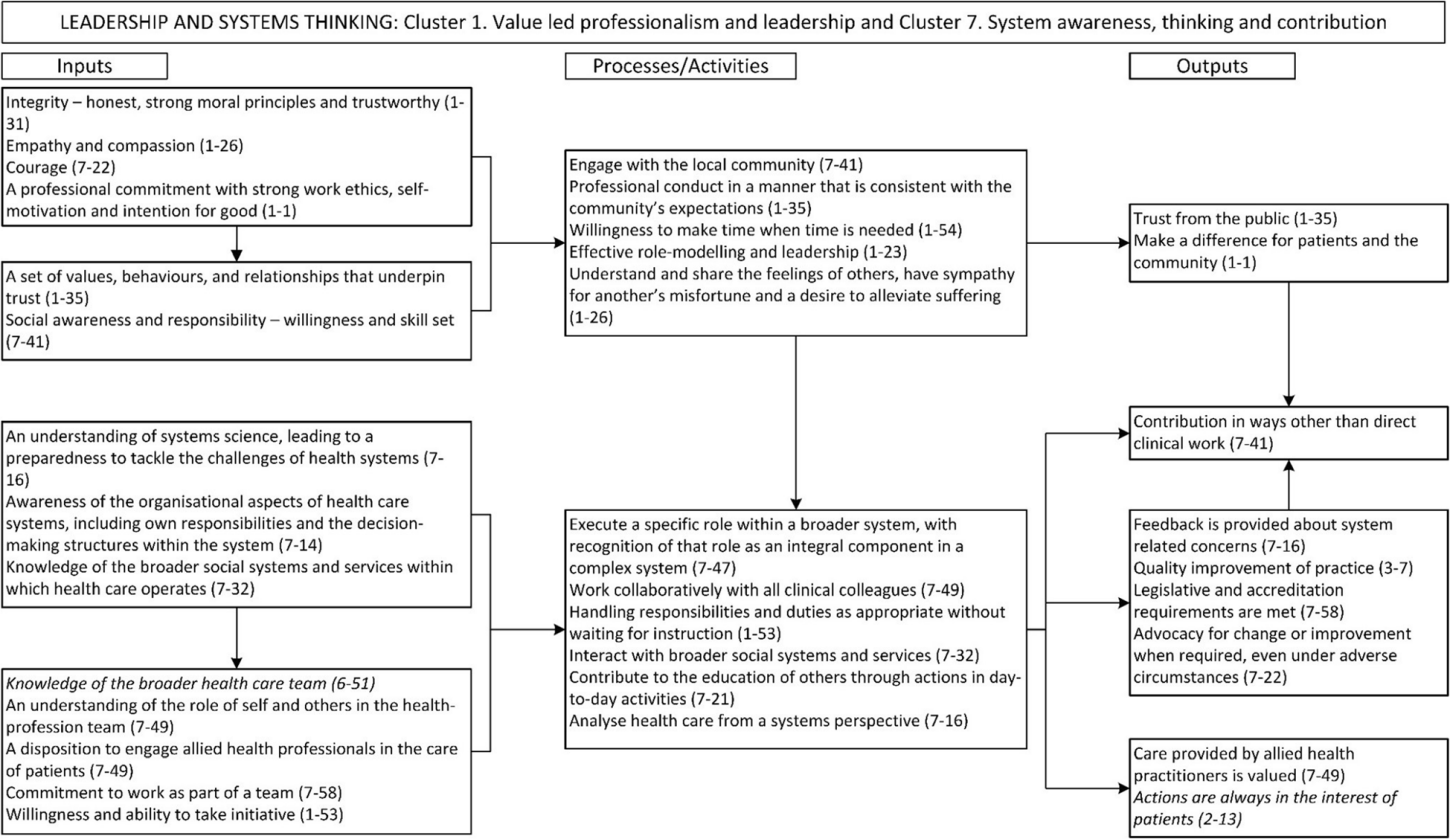
Logic Model. Leadership and systems thinking

Three logic models, one for each domain, were identified, with numbering indicating the cluster and statement number (e.g. 7–22 comes from cluster 7, statement 22, as designated in Additional file 2). The models demonstrate the integration of clusters into domains or meta-competencies, with statements which spanned across domains are highlighted in italics. The model for Leadership and systems thinking is shown (Fig. 3), the other two are available in the Additional file 1 (Item 3).

### Junior doctor ratings for importance and preparedness

#### Cluster rankings and pattern matches

**Fig. 4:**
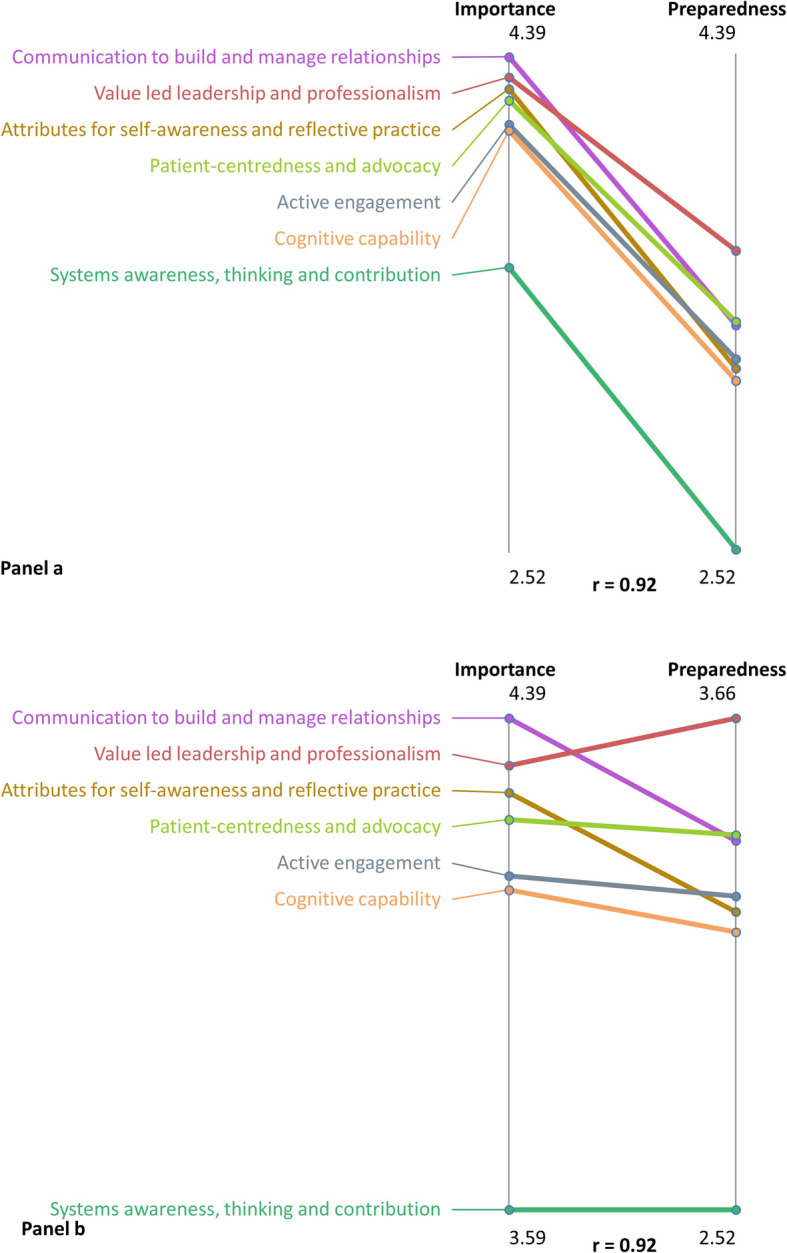
Pattern matches for Importance vs Preparedness. Panel **a**: Unadjusted axes. Panel **b**: Adjusted axes

Ratings with relative rankings for each cluster are found in Table 3 and visually demonstrated in the pattern matches (Fig. 4, Data - participant ratings provided). All items rated above the midpoint on the Likert scale for importance, indicating that none were perceived as not important. Ratings for preparedness for practice were lower than those for importance across each cluster (Fig. 4, panel a). Closer examination is possible by adjusting the axes to show the relative positions of each cluster from highest to lowest ranked (Fig. 4, panel b). This shows that the relative discrepancy between importance and preparedness is greatest for Cluster 5: Communication to Build and Manage Relationship (most important but third highest in preparedness) and Cluster 2: Attributes for Self-awareness and Reflective Practice (third most important but fifth highest in preparedness). Value-led leadership and professionalism was rated third highest for importance, however, was the cluster for which respondents felt most prepared. Systems awareness, thinking and contribution ranked clearly the lowest for importance, however there was an even greater drop in perceived preparedness than other clusters.

**Table 3:** Average rating of importance and preparedness (scale 1-5) for each of the seven clusters

Cluster	Average rating for importance	Average rating for preparedness
1. Value led leadership and professionalism	4.31	3.66
2. Attributes for self-awareness and reflective practice	4.27	3.21
3. Cognitive capability	4.11	3.16
4. Active engagement	4.13	3.24
5. Communication to build and manage relationships	4.39	3.37
6. Patient-centredness and advocacy	4.23	3.39
7. Systems awareness, thinking and contribution	3.59	2.52

Pairwise comparisons were conducted (Mann-U Whitney) to determine whether significant differences between clusters were apparent and are available in the Additional file 1 (Item 4). Cluster 7 was found to be significantly differently rated than the other clusters across both ratings (*p*-values all < 0.02).

#### Go-zones for individual statements

**Fig. 5:**
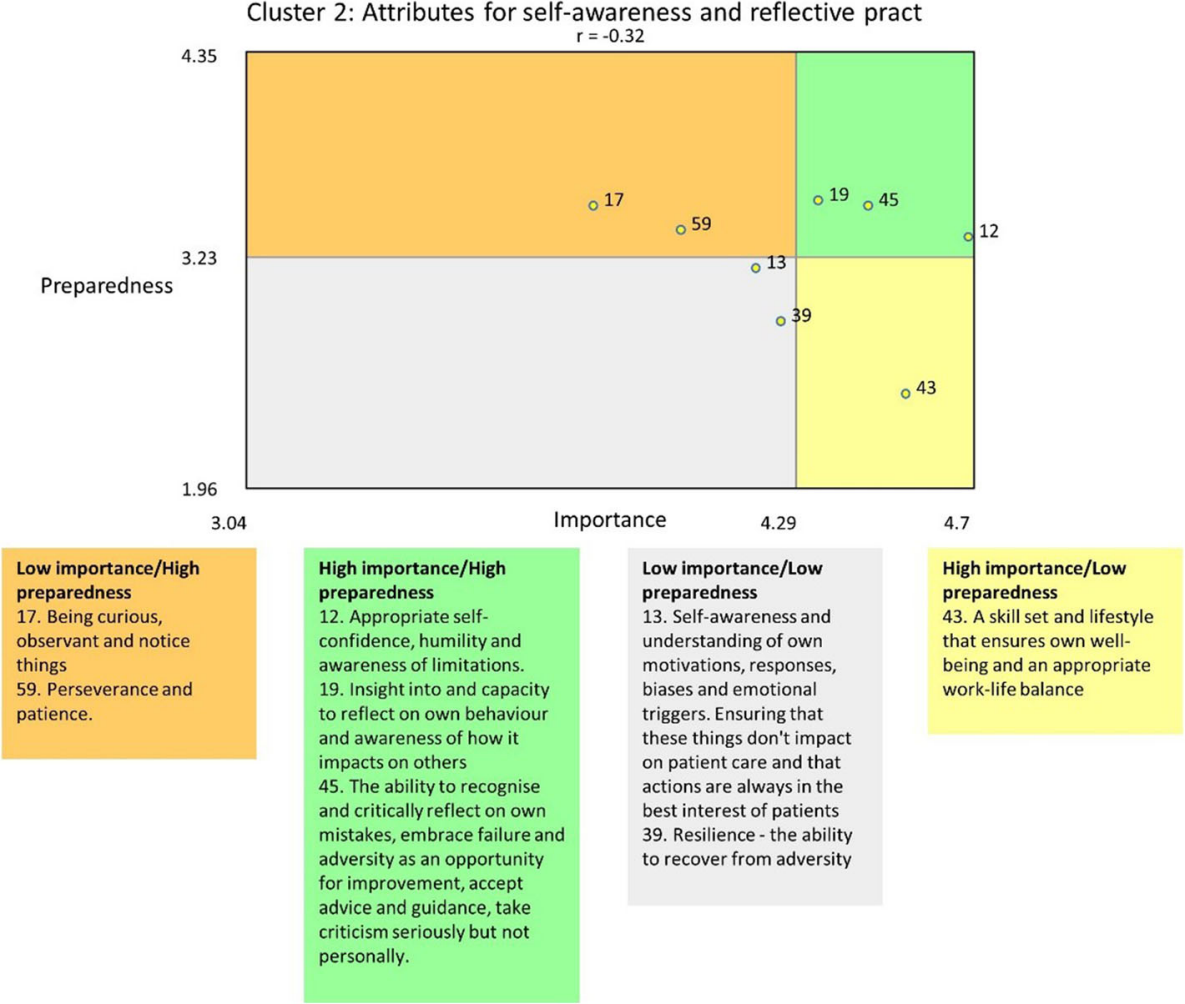
Go-zone Cluster 2. Attributes for self-awareness and reflective practice

All but one go-zones had positive correlations of between 0.45 and 0.77, indicating that items that respondents felt were more important, they were generally more prepared for (Additional file 1, Item 5). Cluster 2 however, showed a negative correlation between responses for Importance and Preparedness (Fig. 5), with one notable statement (43. A skill set that ensures own well being and an appropriate work-life balance) which rated highly for importance but low for preparedness. This is one of seven statements which rated above the overall average for importance and below the average for preaparedness (Table 4). Of note three of these statements (4, 38 and 39), while distinct, do incorporate similar competencies relating to managing interpersonal relationships and conflict.

**Table 4:** Statements which were rated above average for Importance and below average for Preparedness

Statement no.	Statement detail	Importance	Preparedness
Cluster 2 Statement 13	Self-awareness and understanding of own motivations, responses, biases and emotional triggers. Ensuring that these things don’t impact on patient care and that actions are always in the best interest of patients	4.20	3.17
Cluster 2 Statement 39	Resilience - the ability to recover from adversity	4.26	2.88
Cluster 2 Statement 43	A skill set and lifestyle that ensures own well-being and an appropriate work-life balance	4.54	2.49
Cluster 3 Statement 55	Ability for decisive action by assessing relevant information, putting this into perspective of other considerations, weighing up the risk and benefit and acting accordingly	4.32	3.16
Cluster5 Statement 4	Skills in negotiation and conflict resolution, including the ability to challenge in a non-confrontational manner and to view conflict as a source of learning and innovation	4.37	2.87
Cluster 5 Statement 38	Being able to manage differences in agenda between members of the health team, including the patient	4.20	2.73
Cluster 7 Statement 49	The ability to work collaboratively with all clinical colleagues, which includes: an understanding of the role of self and others in the health professional team; a disposition to engage allied health professionals in the care of patients and value the care that is provided by allied health; and taking on a coordinating leadership role where appropriate	4.18	2.89

## Discussion

Through participatory concept mapping, we have developed a conceptual model of attributes and generic competencies that are required for doctors to contribute effectively in modern health-care systems. Participatory Group Concept Mapping [34] allowed the harnessing the views and experience of multiple stakeholder groups, all of whom have regular contact with health-care systems. A shared representation of these requirements resulted in a seven-cluster Concept Map of Attributes and Generic Competencies, representing 60 attributes/competencies. Seven key areas were identified: value-led leadership and professionalism; attributes for self-awareness and reflective practice; cognitive capability; active engagement; communication to build and manage relationships; patient-centredness and advocacy; and systems awareness, thinking and contribution. On examination, the statements could be transformed into a logic model of inputs (pre-requisite attributes), processes and activities (applied competencies), and outputs that can contribute to an optimal health-care. This empirically derived model represents the integrated views of a range of stakeholders. Through transformation into a logic model links between these elements are identified. The explicit demonstration of how attributes and competencies are incorporated into practice through inputs, processes and activities, leading to desirable outputs provides educators with a translational blueprint upon which to map activities and ensure curricula opportunities to develop and demonstrate relevant behaviours. The logic model transformation highlights a clear distinction between attributes identified as inputs in the logic models, and behaviours identified as processes and activities.

On examination, it is apparent that the attributes and competencies identified are transferable professional skills, required across a range of professional contexts. For example, the pre-requisite attributes (inputs) and activities (processes) which lead to trust, making a difference and contributing to community, ensuring quality, and advocating for change (all outcomes), are desirable attributes across many professions. Achieving rapport, possessing excellent communication skills, and achieving respectful relationships with patients/clients are relevant across the professional landscape, as are the cognitive skills of learning, decisive action, reflection and managing uncertainty. This affirms that we have achieved our objective of highlighting attributes and skills which are not *specific to* the clinical setting, despite being *important in* the clinical setting. The notion of generic skills in education is not new [54], however differentiating generic skills from disciplinary knowledge can be challenging, and finding a way for different disciplines to interpret the skills in their context remains important [55].

CanMEDS [56] is one of the most cited competency frameworks to guide medical education and the attributes and generic competencies identified in this research are visible throughout CanMEDS. However, our deliberate strategy to make explicit what is often tacit, by challenging those contributing to the study to focus attention solely on attributes and generic competencies, enabled us to detail a rich behavioural conceptualization of these aspects of being a doctor. Mapping of the outcomes from this study to the CanMEDS framework (as example, an exercise which could have been done with other frameworks also) determined that items in our Concept Map of Attributes and Generic Competencies are behaviourally anchored and attributional, that is, they describe how the competencies in CanMEDS can be achieved through desirable attributes and their application in practice. Further, the cluster organisation is distinct from the CanMEDS categories, indicating for example that CanMEDS’ ‘Doctor as Collaborator’ incorporates attributes and competencies from five clusters: attributes for self-awareness and reflective practice; active engagement; communication to build and manage relationships; patient-centredness and advocacy; and systems awareness, thinking and contribution (Additional file 3). Emphasized in our research are behaviors that have been identified by stakeholders who work in or engage with health-care systems, collectively in multiple ways, bringing a practicality to the outcomes of the research.

Identifying post-graduate trainees with the desired attributes provides a challenge for specialty program selection, with academic parameters heavily weighted at the expense of holistic and equitable selection based on a broader set of attributes and generic competencies [57]. A shift in paradigm towards evidence of generic as well as discipline specific competencies for selection is in process [57], however this shift requires concordance across the education continuum in identifying, validating, and ensuring the development of such the desired competencies.

Fraser and Greenhalgh urge that we move beyond educating for competency, to educating for capability: “the ability to adapt to change, generate new knowledge, and continuously improve performance” ([58], p.799). The product of this research highlights requirements of doctors that will lead to capability; medical educationalists are entrusted to provide opportunities for students to acquire, practice and master. Understanding these enables mapping of curricula and development of innovative pedagogies and opportunities required for capability and to transform the medical workforce to meet current and future health-care needs [58, 59]. The design of educational programs must adapt to ensure that future doctors are equipped for the challenges of modern health-care. This need is exemplified by a lens of complexity science on health-care systems [60] which views health-care as built around multiple interacting systems [61], requiring us to treat health-care improvement as a learning system where participants, including doctors, are attuned to systems features and can build momentum for change [62].

In medical education, students need to be provided with opportunities to develop and practice generic skills [32], and explicating them can aid this process. The Carnegie Foundation for the Advancement of Teaching in 2010 identified the need for innovation in, and new conceptions of, medical education [8]. The report offered recommendations for the types of curricular activities that could support the integration of contemporary competencies, including activities such as longitudinal connection with patients, opportunities to experience the broader professional roles of doctors, interprofessional education and teamwork, locating education in settings other than hospitals, offering feedback and reflective opportunities, engaging learners in initiatives focused on population health, quality improvement and patient safety, and more [8]. Further work in identifying how to link pedagogy with the acquisition of desirable attributes and competencies is needed, however it is apparent that a wide range of opportunities will be required.

The need to place greater weight on non-clinical competencies in medical education has been identified [63] as has a need for incorporation of systems sciences [64, 65]. A small sample of newly graduated doctors rated each of the 60 attributes and competencies according to their perceived importance and their preparedness to perform in the way described after graduation. This group identified the cluster of statements relating to ‘systems awareness, thinking and contribution’ as relatively less important, and for which they were less prepared, compared with all other clusters. The finding is consistent with experiences in two medical schools in the US [66] where systematic introduction of a health-systems curriculum has been challenging on several fronts, one of the most notable challenges being mixed receptivity of students. The authors identified tensions in students’ perception of their professional roles, not seeing systems reform as something which is important for them or feeling powerless to contribute [66]. Further, there are seven important items which were rated highly for importance, and lowly for preparedness. Although a small sample, this illustrates how the conceptual model can be used to inform medical education in a local setting by identifying priority areas to be better integrated into education programs.

We acknowledge that there are limitations to this study. It is relatively small, undertaken in a limited geographic region and its external validity has not been demonstrated. However, mapping of the constructs and domains to existing competency frameworks provides validation of the content of the model broadly, with this study a distinctive extension of existing frameworks due to the participatory concept mapping methodology it uses and the limited focus on attributes and generic competencies only. Ratings were completed by one distinct population with a small sample which was more heterogenous in respect to years post-graduation than we planned. This exposed a weakness in our recruitment processes. However we believe that despite this there is an important outcome demonstrated relating to the different response to Cluster 7 for both importance and preparedness compared with other clusters. The data demonstrate how this methodology can be used locally to identify priorities and to identify differences between stakeholder groups. Such work can lead to important consensus building activities in developing local priorities. Further work is required to determine how doctors acquire the attributes and competencies identified, and how they are enacted in clinical practice to lead to positive outcomes for patients and for the health-care systems. This can then inform the necessary educational opportunities that will lead to their acquisition.

## Conclusion

Changes in health-care systems and health-care delivery require a focus on attributes and generic competencies of doctors to ensure effective contribution in complex health care systems. This research highlights and operationalizes this set of competencies as viewed by participants in the health-care system. Three overarching domains were identified: leadership and systems thinking; learning and cognitive processes; and interpersonal capability. It is broadly recognised that pre-vocational medical education must continue to develop learning opportunities to address the acquisition of a contemporary skill set. The logic model has provided evidence of a link between attributes identified in the research, and generic competencies, that lead to desirable outcomes. Explicating desirable attributes and generic competencies will allow future investigation into which are currently well achieved and which are not in differing contexts, thereby informing pedagogical innovation.

## Supplementary Information

**Additional file 1.** Additional data and information as described in the manuscript.

**Additional file 2.** Full list of 60 statements sorted into 7 clusters – Concept Map of Attributes and Generic Competencies.

**Additional file 3.** Additional information as described in the manuscript.

**Additional file 4.** Sorting dataset presented as a similarity matrix.

**Additional file 5.** Rating dataset.

## Data Availability

There are three datasets generated from the study. The similarity matrix (representative of sorting data) and participant rating data have been made available. Data representing statements generated from brainstorming was iteratively edited and is difficult to represent simply. The authors are willing to provide this upon request, given a detailed explanation of the process undertaken is required to make sense of the spreadsheets on which they are stored. Access to datasets can be obtained by contacting the corresponding author.

## Abbreviation

GCM: Group concept mapping
