# Supplementary material for: Attributes and generic competencies required of doctors: findings from a participatory concept mapping study
Source: BMC Health Serv Res. 2021 Jun 7;21:560. doi: 10.1186/s12913-021-06519-9 (PMC8186188; doi:10.1186/s12913-021-06519-9)
Supplement: Supplementary file 1 — Additional file 1. Additional data and information as described in the manuscript. [file 12913_2021_6519_MOESM1_ESM.pdf]

Online appendix 1

Item 1. Instructions for and flow of data synthesis

Instructions for statement synthesis.

For each statement:

- Confirm that it is either an attribute or **non-clinical** skill - a non-clinical skill is defined as one which is applied in a clinical context, but is not unique to medicine and is a transferable professional skill.
- Does it require further explanation?
- Is it relevant for doctors?
- Is there too much tied up within one statement –i.e. the notions contained within it are potentially exclusive and need to be separated?
- Are there any statements which in your view could be combined?

Figure. *Flowchart detailing statement synthesis*

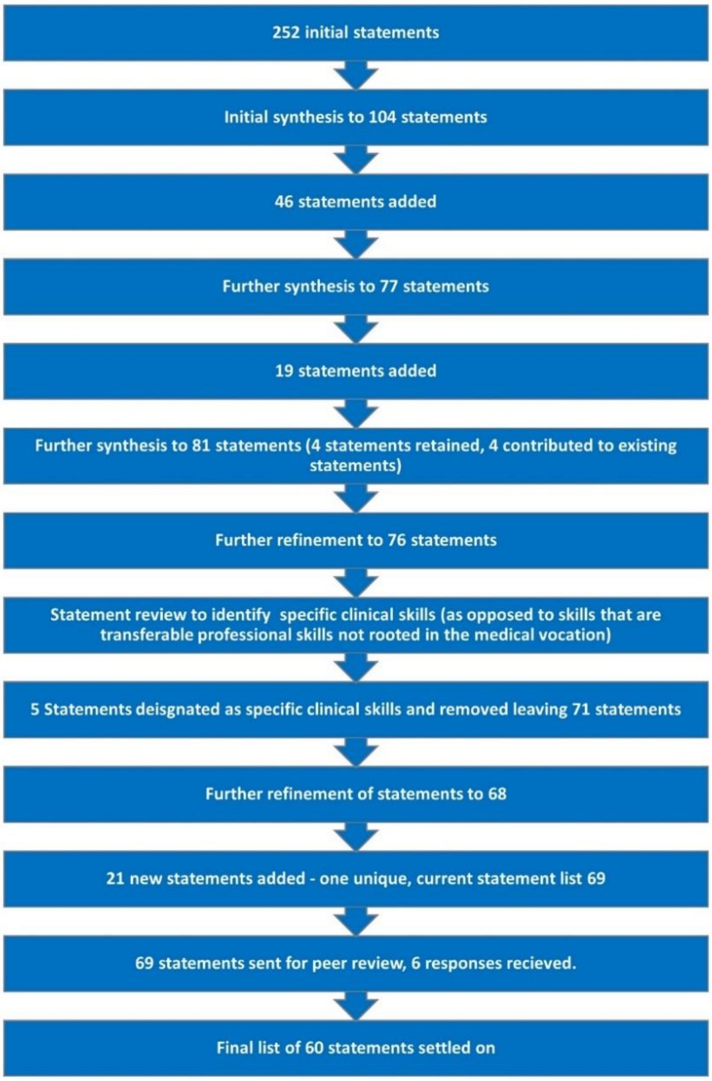

Item 2. Details of four statements which were moved to an adjacent cluster

**Statement (number and content)**	**Original Cluster**	**Final Cluster**
20. Metacognition: Higher order clarity of thought processes with control over cognitive processes through understanding and analysis	Cluster 2. Attributes for self –awareness and reflective practice	Cluster 3. Cognitive capability
56. Ability for decisive action by assessing relevant information, putting this into perspective of other considerations, weighing up the risk and benefit and acting accordingly	Cluster 2. Attributes for self –awareness and reflective practice	Cluster 3. Cognitive capability
33. Literacy in computers and modern technologies, with the ability to efficiently use technology to facilitate communication	Cluster 1. Value-led professionalism and leadership	Cluster 5. Communication to build and manage relationships
22. Courage to advocate for change or improvement when required even under adverse circumstances	Cluster 3. Cognitive capability	Cluster 7. Systems awareness, thinking and contribution

Item 3. Logic transformation models

Logic Model: Leadership and systemic thinking (numbers in parenthesis refer to cluster - statement numbers from the conceptual map

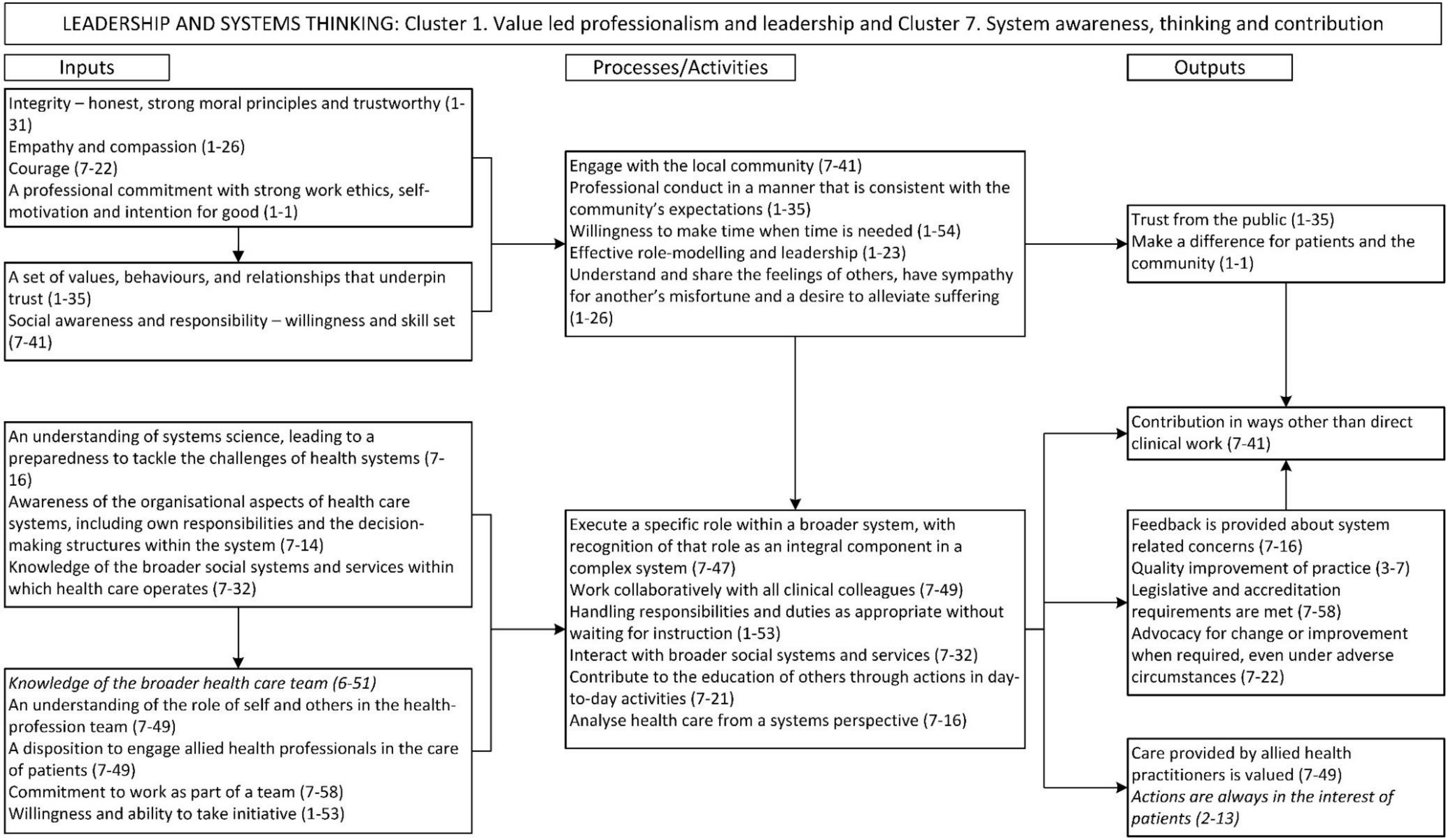

Figure. Logic model: Learning and cognitive processes. Numbers in parenthesis refer to cluster - statement numbers from the conceptual map

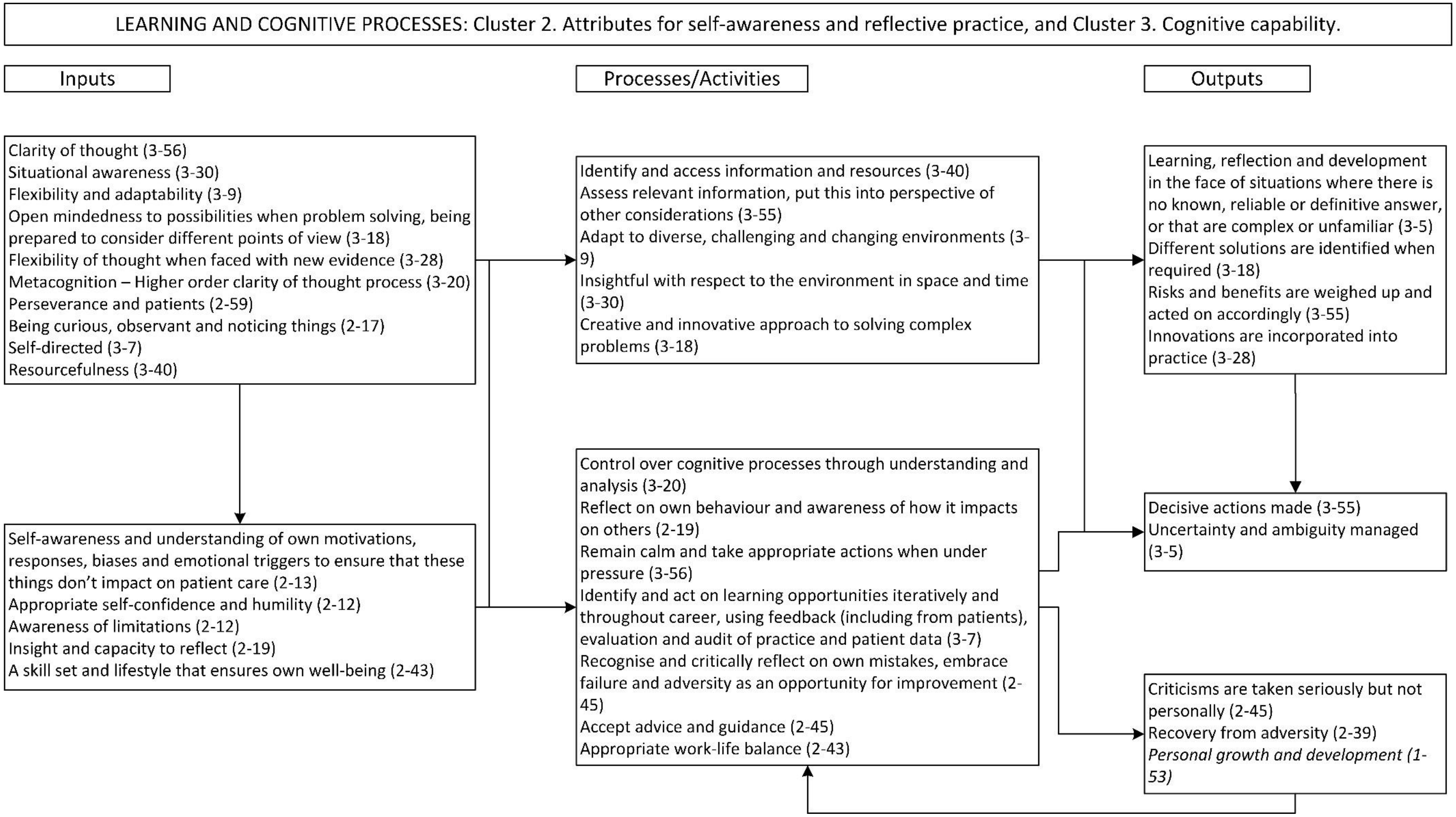

Figure. Logic model: Interpersonal capability. Numbers in parenthesis refer to cluster-statement numbers from the conceptual map

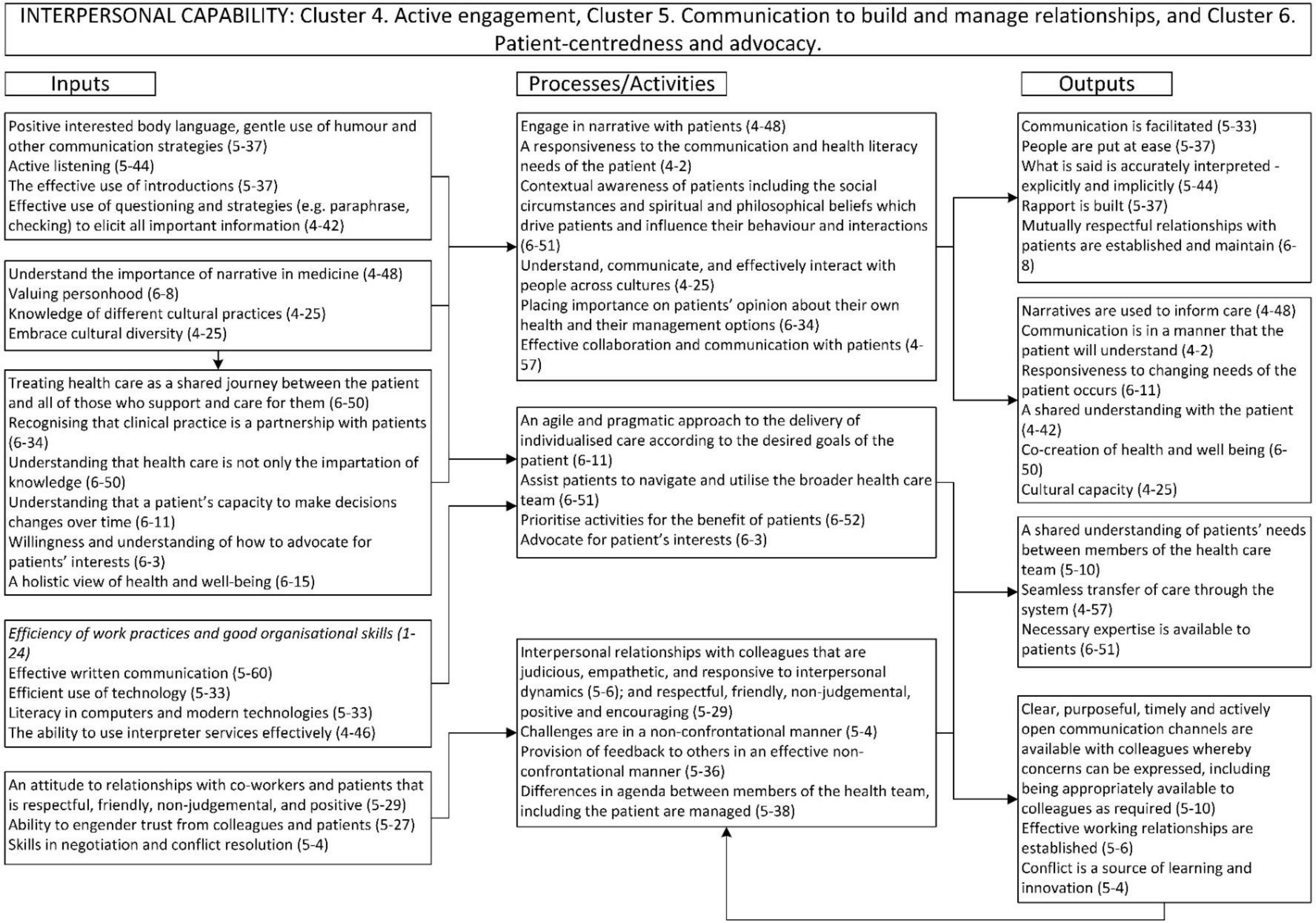

Item 4

Comparison of cluster ratings

Averages were calculated for illustrative purposes for each cluster and clusters ranked according to least-most important, and least-most prepared. Given the non-parametric nature of the rating scale, clusters were also sorted according to perceived importance and preparedness according to mean ranks, identified using the Kruskal-Wallis Test, and significance of the difference across the seven clusters was determined. A visual 'pattern match' was produced which demonstrates for each cluster, relative importance and preparedness, allowing the easy identification of clusters which are perceived as more or less important, and how this relates to perception of preparedness.

Mann-Whitney U tests for non-parametric data were used to conduct pairwise comparisons of clusters for importance and preparedness, utilising ratings of all statements within each cluster. In order to represent the effect size of pairwise comparisons with a p-value of less than 0.05, two analyses were undertaken. 1) Probability of superiority (calculated using the U-statistic from the Mann-Whitney U test and the sample size from each of the two samples: $\hat{\ }_{a.b}$ = U/(axb)), and 2) Effect size correlation (calculated using the *z*-score and sum of the two sample sizes (r=$z/\sqrt{(a+b)}$) [1]. Practical significance of detected differences in pairwise comparisons was determined using an effect size of > 0.4 and correlation of < 2 [2].

Cluster 7 (Systems awareness, thinking and contribution) was ranked significantly lower than the other 6 clusters, with a moderate effect size for both importance and preparedness. The only other significant difference in pairwise comparisons was that Cluster 1 (Value-led professionalism) was rated more highly than Cluster 3 (Cognitive capability) for preparedness.

Pairwise comparisons for each cluster rating of importance and preparedness, including effect size correlation and probability of superiority

		Importance**		Preparedness**	
Cluster comparison	P-value*	Effect size correlation (r)	Probability of superiority ($\hat{p}_{a.b}$)	Effect size correlation (r)	Probability of superiority ($\hat{p}_{a.b}$)

1 v 2	0.48	0.03	0.48	0.18	0.40
1 v 3	0.02	0.10	0.45	0.24	0.37
1 v 4	0.33	0.04	0.48	0.19	0.39
1 v 5	0.05	0.08	0.46	0.13	0.43
1 v 6	0.87	0.01	0.46	0.12	0.43
1 v 7	<0.01	0.32	0.35	0.49	0.22
2 v 3	<0.01	0.13	0.43	0.07	0.46
2 v 4	0.12	0.07	0.46	0.03	0.48
2 v 5	0.24	0.05	0.48	0.04	0.48
2 v 6	0.41	0.04	0.48	0.06	0.47
2 v 7	<0.01	0.34	0.31	0.35	0.31
3 v 4	0.31	0.05	0.47	0.04	0.48
3 v 5	<0.01	0.18	0.40	0.11	0.44
3 v 6	0.04	0.09	0.45	0.13	0.43
3 v 7	<0.01	0.23	0.37	0.27	0.35
4 v 5	0.01	0.12	0.43	0.07	0.46
4 v 6	0.42	0.04	0.48	0.09	0.45
4 v 7	<0.01	0.26	0.35	0.30	0.33
5 v 6	0.04	0.09	0.45	0.01	0.49
5 v 7	<0.01	0.39	0.28	0.37	0.29
6 v 7	<0.01	0.31	0.33	0.40	0.28

*Mann U Whitney

**Important differences are those with r > 0.2 and $(\hat{p}_{a.b}) < 0.4$

1. Rosnow RL, Rosenthal R, Rubin DB: Contrasts and Correlations in Effect-Size Estimation. Psychol Sci 2000, 11(6):446-453.
2. Lipsey MW, Puzio K, Yun C, Hebert MA, Steinka-Fry K, Cole MW, Roberts M *et al*: Translating the Statistical Representation of the Effects of Education Interventions into More Readily Interpretable Forms. National Center for Special Education Research 2012.

Item 6. Go-zones for each cluster.

Go-zones are visual representation of rating data. Each statement is represented in its respective cluster go-zone, with the average rating for importance plotted against the average rating for preparedness. Quadrants are represented as below:

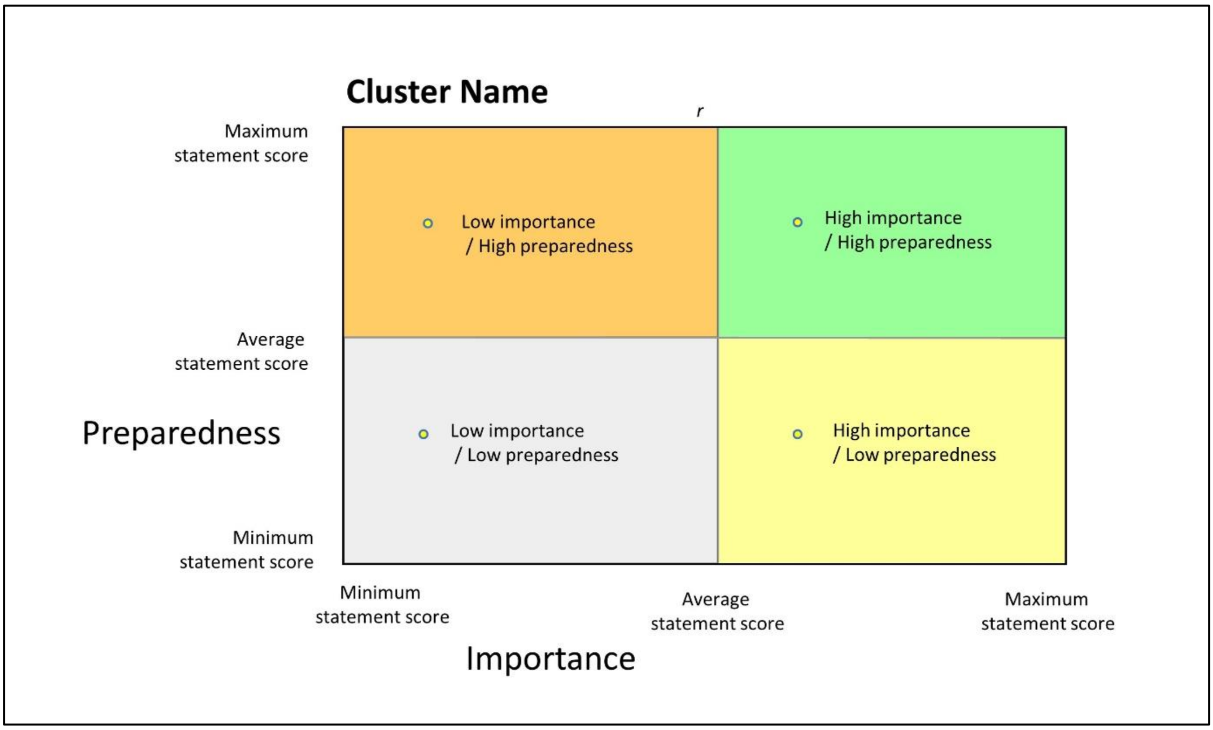

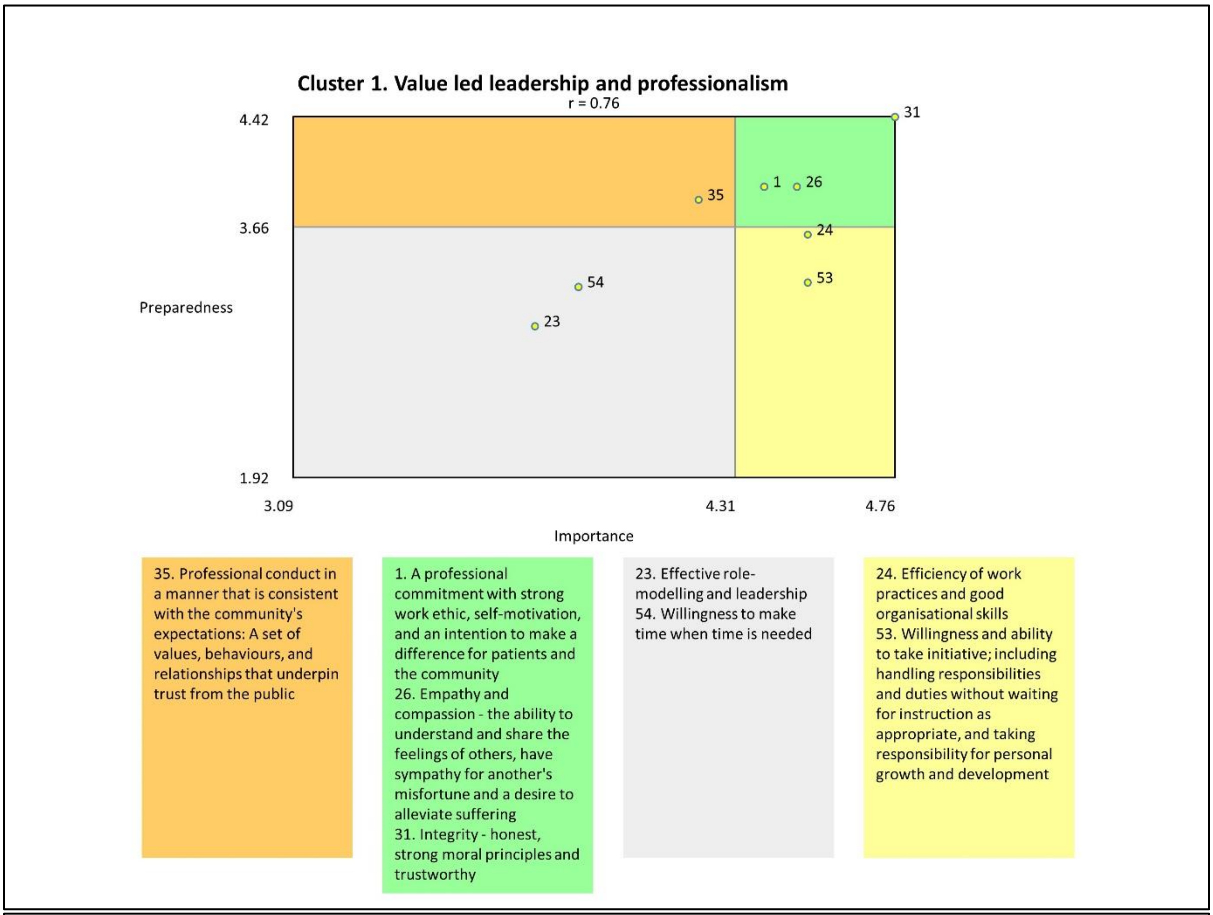
Cluster 1. Value led leadership and professionalism
r = 0.76
4.42
3.66
1.92
Preparedness
3.09
4.31
4.76
Importance
31
1
26
35
24
53
54
23
35. Professional conduct in a manner that is consistent with the community's expectations: A set of values, behaviours, and relationships that underpin trust from the public
1. A professional commitment with strong work ethic, self-motivation, and an intention to make a difference for patients and the community
26. Empathy and compassion - the ability to understand and share the feelings of others, have sympathy for another's misfortune and a desire to alleviate suffering
31. Integrity - honest, strong moral principles and trustworthy
23. Effective role-modelling and leadership
54. Willingness to make time when time is needed
24. Efficiency of work practices and good organisational skills
53. Willingness and ability to take initiative; including handling responsibilities and duties without waiting for instruction as appropriate, and taking responsibility for personal growth and development

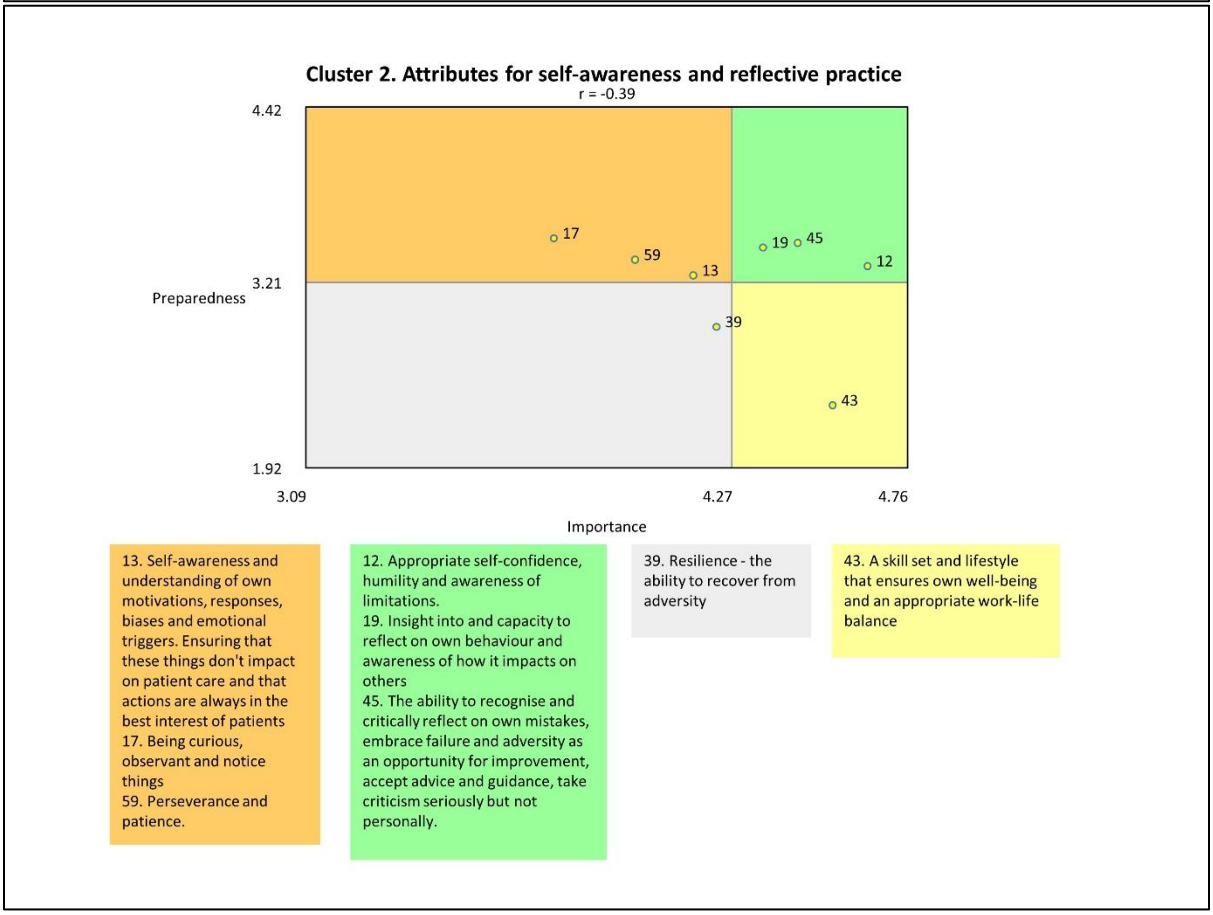
Cluster 2. Attributes for self-awareness and reflective practice
r = -0.39
4.42
3.21
1.92
Preparedness
3.09
4.27
4.76
Importance
17
59
13
19
45
12
39
43
13. Self-awareness and understanding of own motivations, responses, biases and emotional triggers. Ensuring that these things don't impact on patient care and that actions are always in the best interest of patients
17. Being curious, observant and notice things
59. Perseverance and patience.
12. Appropriate self-confidence, humility and awareness of limitations.
19. Insight into and capacity to reflect on own behaviour and awareness of how it impacts on others
45. The ability to recognise and critically reflect on own mistakes, embrace failure and adversity as an opportunity for improvement, accept advice and guidance, take criticism seriously but not personally.
39. Resilience - the ability to recover from adversity
43. A skill set and lifestyle that ensures own well-being and an appropriate work-life balance

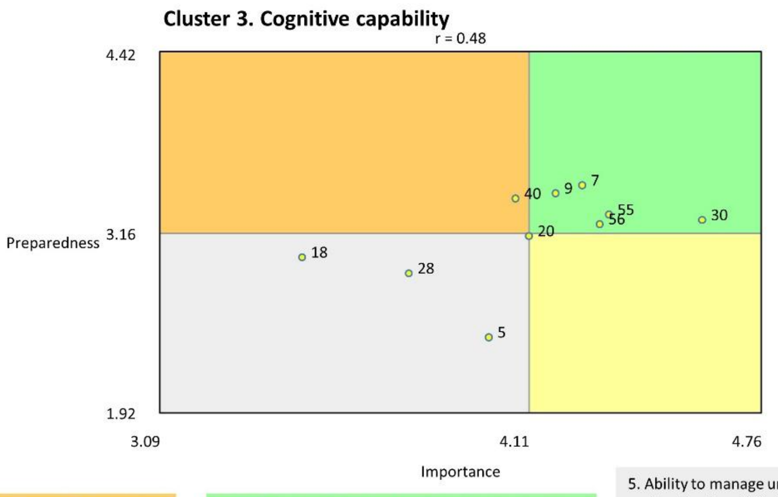

40. Resourcefulness: ability to identify and access information and resources to aid clinical care.

7. Self-directed ability to identify and act on learning opportunities iteratively and throughout career, including the use of feedback (including from patients), evaluation and audit of practice and patient data as an impetus for quality improvement of practice
9. Flexibility and adaptability to adapt to diverse, challenging and changing environments
30. Situational awareness: insightful with respect to the environment in space and time.
55. Ability for decisive action by assessing relevant information, putting this into perspective of other considerations, weighing up the risk and benefit and acting accordingly
56. Clarity of thought and ability to remain calm and take appropriate actions when under pressure

5. Ability to manage uncertainty and ambiguity: the ability to learn, reflect and develop in the face of situations where there is no known, reliable or definitive answer; or that are complex or unfamiliar.
18. Creative and innovative approach to solving complex problems. Being open minded to possibilities when problem solving, be prepared to consider different points of view and to look for different solutions when required.
20. Metacognition: Higher order clarity of thought processes with control over cognitive processes through understanding and analysis.
28. Flexibility of thought when faced with new evidence and incorporate innovations into practice

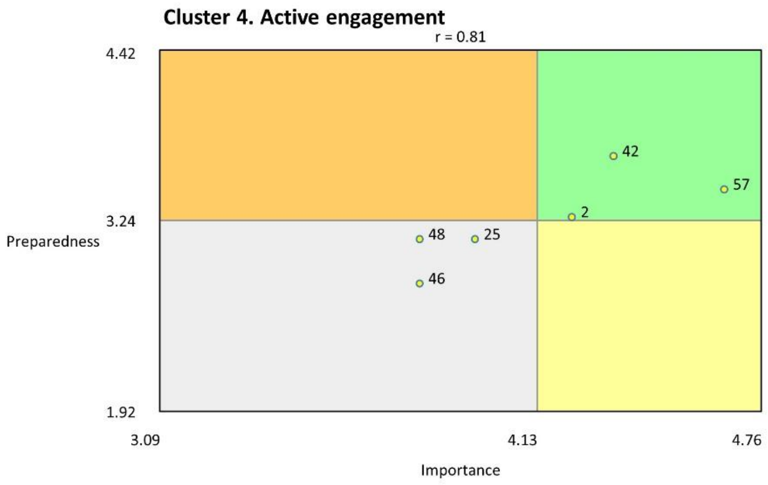

2. A responsiveness to the communication and health literacy needs of the patient, including adaptability to ensure communication is in a manner that patients will understand
42. The effective use of questioning and strategies (e.g. paraphrasing, checking) to elicit all important information and ensure shared understanding with the patient
57. Ensure, through effective collaboration with colleagues and communication with patients, that there is a seamless transfer of care through the system

25. Embrace cultural diversity and ensure cultural capacity, including the ability to understand, communicate with, and effectively interact with people across cultures, and knowledge of different cultural practices.
46. The ability to use interpreter services effectively
48. Understand the importance of narrative in medicine; to engage in narrative with patients and use these narratives to inform the care provided

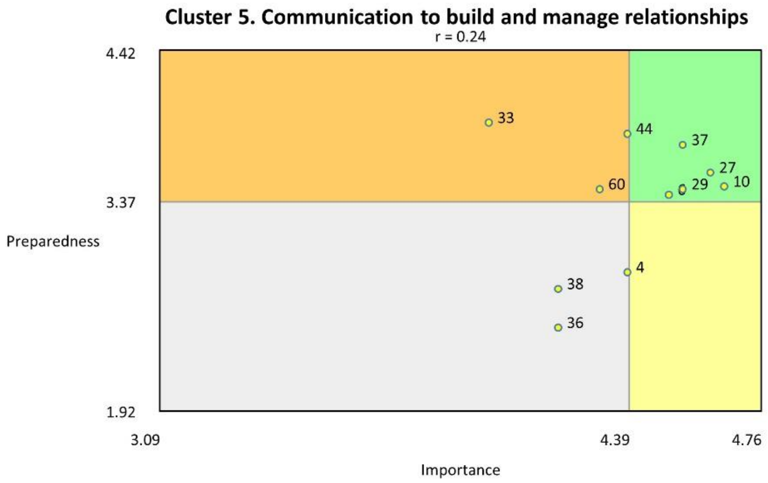

33. Literacy in computers and modern technologies, with the ability to efficiently use technology to facilitate communication
44. The ability to listen and accurately interpret what is being said (explicitly and implicitly)
60. Effective written communication skills

6. An approach to interpersonal relationships with colleagues that is judicious, empathetic, and responsive to interpersonal dynamics to ensure effective working relationships
10. Clear, purposeful, timely and actively open communication channels with colleagues ensuring a shared understanding of patient's needs between members of the health care team and that concerns can be expressed, including being appropriately available for consultation with colleagues as required
27. Ability to engender trust from colleagues and patients
29. An attitude and approach to relationships with co-workers and patients that is respectful, friendly, non-judgemental, positive and encouraging.
37. Putting people at ease and building rapport through the effective use of introductions to clarify roles, positive interested body language, gentle use of humour, and other mechanisms

4. Skills in negotiation and conflict resolution, including the ability to challenge in a non-confrontational manner and to view conflict as a source of learning and innovation
36. Provide feedback to others in an effective non-confrontational manner when required
38. Being able to manage differences in agenda between members of the health team, including the patient

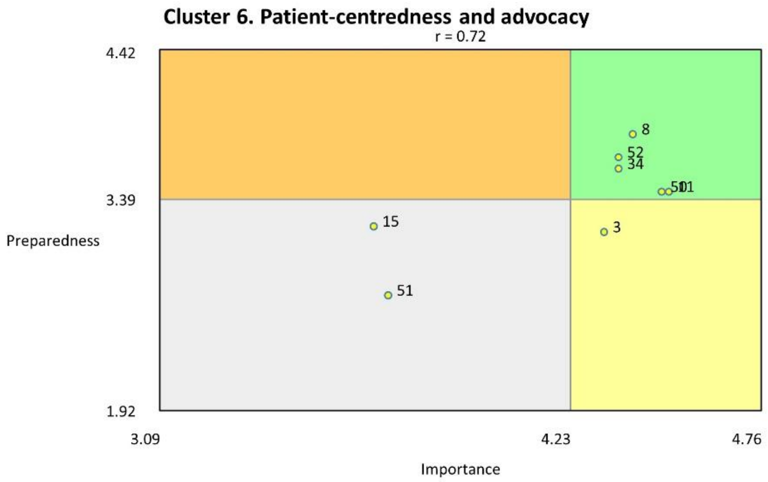

8. Ability to establish and maintain mutually respectful relationships with patients valuing personhood
11. An agile and pragmatic approach to the delivery of individualised care according to the desired goals of the patient. Includes a responsiveness to changing needs of the patient and an understanding that a patient's capacity to make decisions changes over time
34. Recognising that clinical practice is a partnership with patients, and placing importance on patients' opinion about their own health and their management options
50. Treating health care as a shared journey between the patient and all of those who support and care for them, with a willingness and ability to work with patients in order to co-create health and well-being; with this comes an understanding that healthcare is not only the impartation of knowledge.
52. Willingness and ability to prioritise activities for the benefit of patients

15. Contextual awareness of patients including the social circumstances and spiritual and philosophical beliefs, which drive patients and influence their behaviour and interactions, leading to a holistic view of health and well-being.
51. Knowledge and ability to assist patients to navigate and utilise the broader health care team to ensure all necessary expertise is available to them

3. A willingness and understanding of how to advocate for patients' interests

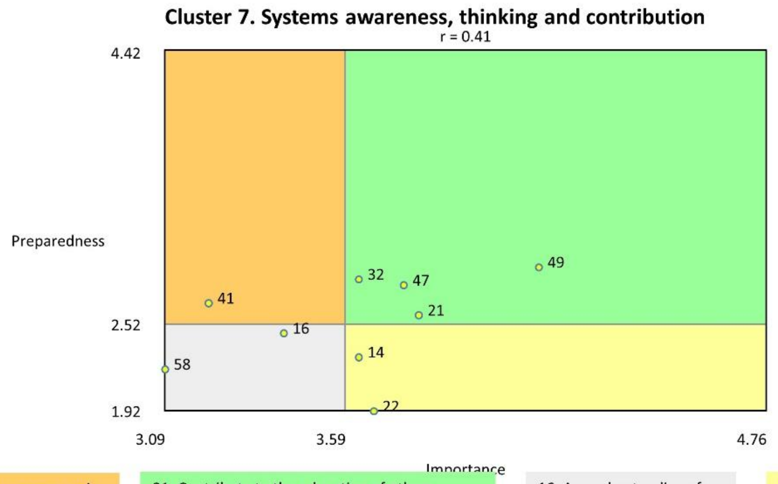

41. Social awareness and responsibility, with a willingness and skill set to engage with the local community and contribute in ways other than direct clinical work

21. Contribute to the education of others through actions in day-to-day activities
32. Knowledge of the broader social systems and services within which health care operates, enabling interaction with them for the benefit of patient care.
47. The ability to execute a specific role within a broader system, with recognition of that role as an integral component of a complex system
49. The ability to work collaboratively with all clinical colleagues, which includes: an understanding of the role of self and others in the health professional team; a disposition to engage allied health professionals in the care of patients and value the care that is provided by allied health; and taking on a coordinating leadership role where appropriate

16. An understanding of systems science, leading to a preparedness to tackle the challenges of health systems including ability to analyse health care from a systems perspective and providing feedback about system related concerns
58. Commitment to work as part of a team to meet legislative and accreditation requirements

14. Awareness of the organisational aspects of health care systems, including own responsibilities and the decision-making structures within the system
22. Courage to advocate for change or improvement when required even under adverse circumstances
