# Supplementary material for: Attributes and generic competencies required of doctors: findings from a participatory concept mapping study
Source: BMC Health Serv Res. 2021 Jun 7;21:560. doi: 10.1186/s12913-021-06519-9 (PMC8186188; doi:10.1186/s12913-021-06519-9)
Supplement: Supplementary file 2 — Additional file 2. Full list of 60 statements sorted into 7 clusters – Concept Map of Attributes and Generic Competencies. [file 12913_2021_6519_MOESM2_ESM.pdf]

Appendix 2. All statements in final cluster groups prefaced by statement number.

Concept Map Statements of Attributes and Generic Competencies for Doctors

**Cluster 1. Value-led professionalism and leadership**	
53	Willingness and ability to take initiative; including handling responsibilities and duties without waiting for instruction as appropriate, and taking responsibility for personal growth and development
1	A professional commitment with strong work ethic, self-motivation, and an intention to make a difference for patients and the community
31	Integrity - honest, strong moral principles and trustworthy
35	Professional conduct in a manner that is consistent with the community's expectations: A set of values, behaviours, and relationships that underpin trust from the public
23	Effective role-modelling and leadership
24	Efficiency of work practices and good organisational skills
54	Willingness to make time when time is needed
26	Empathy and compassion - the ability to understand and share the feelings of others, have sympathy for another's misfortune and a desire to alleviate suffering
**Cluster 2. Attributes for self-awareness and reflective practice**	
43	A skill set and lifestyle that ensures own well-being and an appropriate work-life balance
19	Insight into and capacity to reflect on own behaviour and awareness of how it impacts on others
45	The ability to recognise and critically reflect on own mistakes, embrace failure and adversity as an opportunity for improvement, accept advice and guidance, take criticism seriously but not personally.
39	Resilience - the ability to recover from adversity
12	Appropriate self-confidence, humility and awareness of limitations.
17	Being curious, observant and notice things
13	Self-awareness and understanding of own motivations, responses, biases and emotional triggers. Ensuring that these things don't impact on patient care and that actions are always in the best interest of patients
59	Perseverance and patience
**Cluster 3. Cognitive capability**	
20	Metacognition: Higher order clarity of thought processes with control over cognitive processes through understanding and analysis. (Moved from Cluster 2 – Attributes for self-awareness and reflective practice)
56	Clarity of thought and ability to remain calm and take appropriate actions when under pressure
7	Self-directed ability to identify and act on learning opportunities iteratively and throughout career, including the use of feedback (including from patients), evaluation and audit of practice and patient data as an impetus for quality improvement of practice
18	Creative and innovative approach to solving complex problems. Being open minded to possibilities when problem solving, be prepared to consider different points of view and to look for different solutions when required.

5	Ability to manage uncertainty and ambiguity: the ability to learn, reflect and develop in the face of situations where there is no known, reliable or definitive answer; or that are complex or unfamiliar.
28	Flexibility of thought when faced with new evidence and incorporate innovations into practice
55	Ability for decisive action by assessing relevant information, putting this into perspective of other considerations, weighing up the risk and benefit and acting accordingly (Moved from Cluster 2 – Attributes for self-awareness and reflective practice)
40	Resourcefulness: ability to identify and access information and resources to aid clinical care.
30	Situational awareness: insightful with respect to the environment in space and time.
9	Flexibility and adaptability to adapt to diverse, challenging and changing environments
**Cluster 4. Active engagement**	
2	A responsiveness to the communication and health literacy needs of the patient, including adaptability to ensure communication is in a manner that patients will understand
48	Understand the importance of narrative in medicine; to engage in narrative with patients and use these narratives to inform the care provided
42	The effective use of questioning and strategies (e.g. paraphrasing, checking) to elicit all important information and ensure shared understanding with the patient
46	The ability to use interpreter services effectively
57	Ensure, through effective collaboration with colleagues and communication with patients, that there is a seamless transfer of care through the system
25	Embrace cultural diversity and ensure cultural capacity, including the ability to understand, communicate with, and effectively interact with people across cultures, and knowledge of different cultural practices
**Cluster 5. Communication to build and manage relationships**	
10	Clear, purposeful, timely and actively open communication channels with colleagues ensuring a shared understanding of patient's needs between members of the health-care team and that concerns can be expressed, including being appropriately available for consultation with colleagues as required
37	Putting people at ease and building rapport through the effective use of introductions to clarify roles, positive interested body language, gentle use of humour, and other mechanisms
36	Provide feedback to others in an effective non-confrontational manner when required
6	An approach to interpersonal relationships with colleagues that is judicious, empathetic, and responsive to interpersonal dynamics to ensure effective working relationships
29	An attitude and approach to relationships with co-workers and patients that is respectful, friendly, non-judgemental, positive and encouraging
38	Being able to manage differences in agenda between members of the health team, including the patient
60	Effective written communication skills
33	Literacy in computers and modern technologies, with the ability to efficiently use technology to facilitate communication (Moved from Cluster 1 Value-led professionalism and leadership)

44	The ability to listen and accurately interpret what is being said (explicitly and implicitly)
4	Skills in negotiation and conflict resolution, including the ability to challenge in a non-confrontational manner and to view conflict as a source of learning and innovation
27	Ability to engender trust from colleagues and patients
**Cluster 6. Patient-centredness and advocacy**	
15	Contextual awareness of patients including the social circumstances and spiritual and philosophical beliefs, which drive patients and influence their behaviour and interactions, leading to a holistic view of health and well-being.
34	Recognising that clinical practice is a partnership with patients, and placing importance on patients' opinion about their own health and their management options
3	A willingness and understanding of how to advocate for patients' interests
50	Treating health-care as a shared journey between the patient and all of those who support and care for them, with a willingness and ability to work with patients in order to co-create health and well-being; with this comes an understanding that healthcare is not only the impartation of knowledge
8	Ability to establish and maintain mutually respectful relationships with patients valuing personhood
52	Willingness and ability to prioritise activities for the benefit of patients
11	An agile and pragmatic approach to the delivery of individualised care according to the desired goals of the patient. Includes a responsiveness to changing needs of the patient and an understanding that a patient's capacity to make decisions changes over time
51	Knowledge and ability to assist patients to navigate and utilise the broader health-care team to ensure all necessary expertise is available to them
**Cluster 7. Systems awareness, thinking and contribution**	
21	Contribute to the education of others through actions in day-to-day activities
58	Commitment to work as part of a team to meet legislative and accreditation requirements
14	Awareness of the organisational aspects of health-care systems, including own responsibilities and the decision-making structures within the system
22	Courage to advocate for change or improvement when required even under adverse circumstances (moved from Cluster 4 Active engagement)
47	The ability to execute a specific role within a broader system, with recognition of that role as an integral component of a complex system
32	Knowledge of the broader social systems and services within which health-care operates, enabling interaction with them for the benefit of patient care.
16	An understanding of systems science, leading to a preparedness to tackle the challenges of health systems including ability to analyse health-care from a systems perspective and providing feedback about system related concerns
49	The ability to work collaboratively with all clinical colleagues, which includes: an understanding of the role of self and others in the health professional team; a disposition to engage allied health professionals in the care of patients and value the care that is provided by allied health; and taking on a coordinating leadership role where appropriate
41	Social awareness and responsibility, with a willingness and skill set to engage with the local community and contribute in ways other than direct clinical work
