# Supplementary material for: Attributes and generic competencies required of doctors: findings from a participatory concept mapping study
Source: BMC Health Serv Res. 2021 Jun 7;21:560. doi: 10.1186/s12913-021-06519-9 (PMC8186188; doi:10.1186/s12913-021-06519-9)
Supplement: Supplementary file 3 — Additional file 3. Additional information as described in the manuscript. [file 12913_2021_6519_MOESM3_ESM.pdf]

Mapping CanMEDS to Concept Map

Explanation: This document is provided as explanation of how statements from the Concept Map identified in the research relate to the competencies identified in CanMEDS. There are three sections: 1. Diagram which connects CanMEDS categories with Concept Map clusters; 2. Tables of CanMEDS competencies (left-hand side), with cross-matched statements from the concept map on the (right-hand side). These statements are colour highlighted according to which cluster they came from. Competencies that are greyed out are professionally specific and would not be expected to have a mapped statement from the Concept Map. Those which are highlighted with red text are not covered in the concept map; 3. Each cluster from the Concept Map with statements are provided, identifying statements that are not found in CanMEDS.

In summary, the mapping exercise showed that the vast majority of competencies in CanMEDS can be identified in the Concept Map, and vice versa. It is also noted that the items in the Concept Map are behaviourally anchored and attributional, that is, they describe how the competencies in CanMEDS can be achieved.

1. Diagram representing how statements from the Concept Map interacted with CanMEDS categories

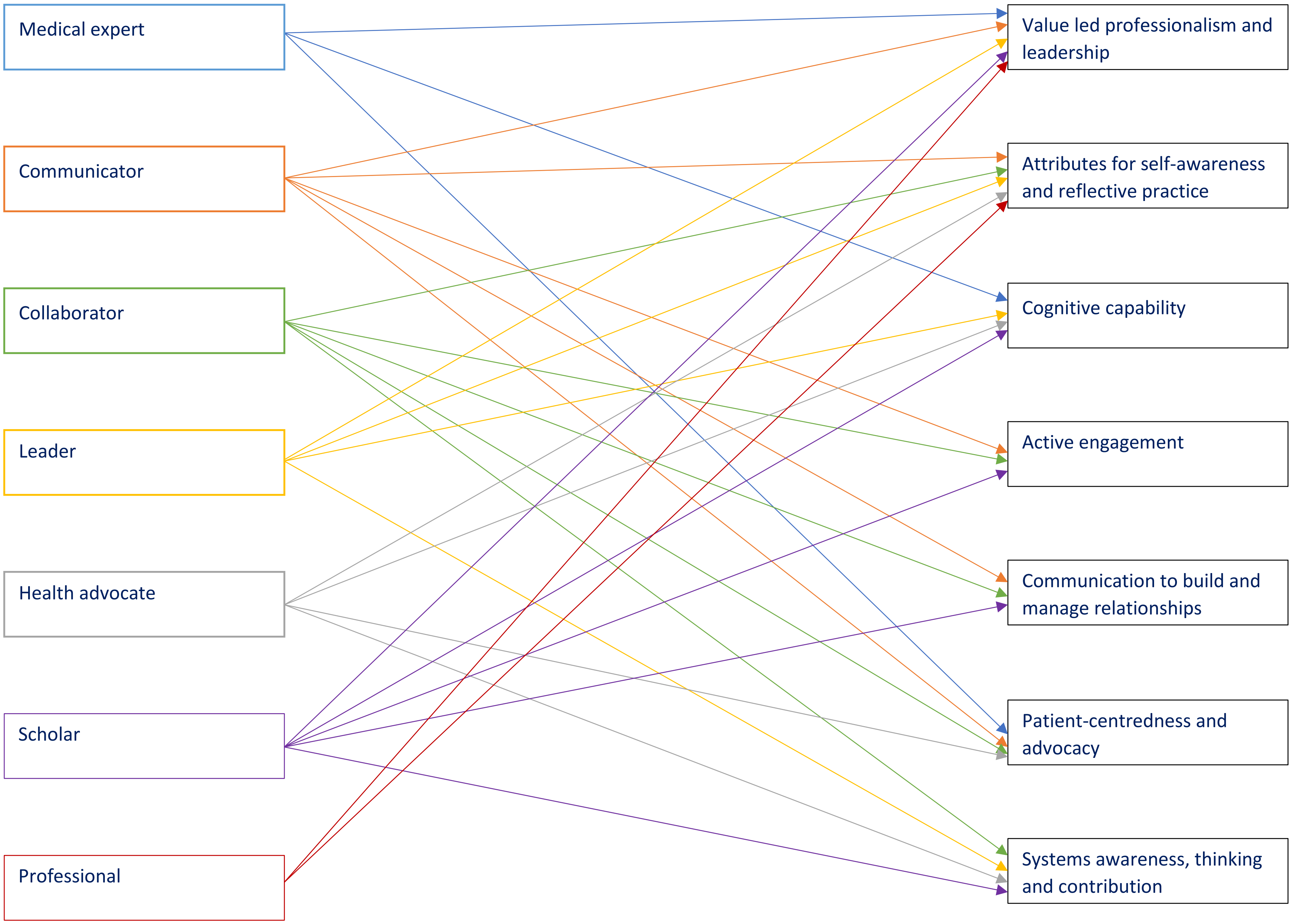

2. Mapping tables

Medical expert

Medical expert incorporates attributes/competencies from Cluster 1, Cluster 3 and Cluster 6. As would be expected there are a number of enabling competencies (9/16) which are clinically specific skills and therefore not found in the conceptual cluster map (CCM). Enabling competency 1.2 (Integrate the CanMEDS Intrinsic Roles into their practice of medicine) is a broad overarching competency which was not identified specifically in the CCM. Resource management was an area found in the Medical Expert role which did not appear in the CCM.

Key competencies

1. Practise medicine within their defined scope of practice and expertise<br>2. Perform a patient-centred clinical assessment and establish a<br>management plan<br>3. Plan and perform procedures and therapies for the purpose of assessment and/or management<br>4. Establish plans for ongoing care and, when appropriate, timely consultation<br>5. Actively contribute, as an individual and as a member of a team providing care, to the continuous improvement of health care quality and patient safety

Enabling competencies

1.1 Demonstrate a commitment to high-quality care of their patients	Cluster 1; statement 1. A professional commitment with strong work ethic, self-motivation, and an intention to make a difference for patients and the community
1.2 Integrate the CanMEDS Intrinsic Roles into their practice of medicine	Integration of roles not identified
1.3 Apply knowledge of the clinical and biomedical sciences relevant to their discipline	*Clinically specific*
1.4 Perform appropriately timed clinical assessments with recommendations that are presented in an organized manner	Cluster 1; statement 24. Efficiency of work practices and good organisational skills
1.5 Carry out professional duties in the face of multiple, competing demands	Cluster 3; statement 9. Flexibility and adaptability to adapt to diverse, challenging and changing environments

1.6 Recognize and respond to the complexity, uncertainty, and ambiguity inherit in medical practice	Cluster 3; statement 5. Ability to manage uncertainty and ambiguity: the ability to learn, reflect and develop in the face of situations where there is no known, reliable or definitive answer; or that are complex or unfamiliar.<br>Cluster 3; statement 18. Creative and innovative approach to solving complex problems. Being open minded to possibilities when problem solving, be prepared to consider different points of view and to look for different solutions when required.
1.2 Prioritize issues to be addressed in a patient encounter	Cluster 3; statement 52. Willingness and ability to prioritise activities for the benefit of patients
2.2 Elicit a history, perform a physical exam, select appropriate investigations, and interpret their results for the purpose of diagnosis and management, disease prevention, and health promotion	*Clinically Specific*
2.3 Establish goals of care in collaboration with patients and their families, which may include slowing disease progression, treating symptoms, achieving cure, improving function, and palliation	Cluster 6; statement 11. An agile and pragmatic approach to the delivery of individualised care according to the desired goals of the patient. Includes a responsiveness to changing needs of the patient and an understanding that a patient's capacity to make decisions changes over time
2.4 Establish a patient-centred management plan	*Clinically Specific*
3.1 Determine the most appropriate procedures or therapies	*Clinically Specific*
3.2 Obtain and document informed consent, explaining the risks and benefits of, and the rationale for, a proposed procedure or therapy	*Clinically Specific*
3.3 Prioritize a procedure or therapy, taking into account clinical urgency and available resources	Resource management not identified in CCM
3.4 Perform a procedure in a skilful and safe manner, adapting to unanticipated findings or changing clinical circumstances	*Clinically Specific*
4.1 Implement a patient-centred care plan that supports ongoing care, follow-up on investigations, response to treatment, and further consultation	*Clinically Specific*

5.1 Recognize and respond to harm from health care delivery, including patient safety incidents	*Clinically Specific*
5.2 Adopt strategies that promote patient safety and address human and system factors	*Clinically Specific*

Communicator

Statements from clusters 1, 2, 4, 5 and 6 were found to correlate with enabling competencies in the Communicator role. Six of 17 items were considered clinically specific, the remainder were able to be correlated with items from the CCM.

CCM items in some cases make more explicit the attributes that will lead to the enabling competencies. Notably item 4.3 (Use communication skills and strategies that help patients and their families make informed decisions regarding their health) is a broad brush statement for which a number of items in the CCM can be mapped to which identify the attributes and competencies that are important in achieving the relationship that will facilitate informed decision making.

1.Establish professional therapeutic relationships with patients and their families<br>2. Elicit and synthesize accurate and relevant information, incorporating the perspectives of patients and their families<br>3. Share health care information and plans with patients and their families<br>4. Engage patients and their families in developing plans that reflect the patient's health care needs and goals<br>5. Document and share written and electronic information about the medical encounter to optimize clinical decision-making, patient safety, confidentiality, and privacy

Enabling competencies

1.1 Communicate using a patient-centred approach that encourages patient trust and autonomy and is characterized by empathy, respect, and compassion	Cluster 1; statement 26. Empathy and compassion - the ability to understand and share the feelings of others, have sympathy for another's misfortune and a desire to alleviate suffering<br>Cluster 5; statement 27. Ability to engender trust from colleagues and patients
1.2 Optimize the physical environment for patient comfort, dignity, privacy, engagement, and safety	*Clinically Specific/implied in other ways (respect etc)*
1.3 Recognize when the values, biases, or perspectives of patients, physicians, or other health care professionals may have an impact on the quality of care, and modify the approach to the patient accordingly	Cluster 2; statement 13. Self-awareness and understanding of own motivations, responses, biases and emotional triggers. Ensuring that these things don't impact on patient care and that actions are always in the best interest of patients

1.4 Respond to a patient's non-verbal behaviours to enhance communication	Picking up on non-verbal communication not overtly identified<br><br>Cluster 5; statement 44. The ability to listen and accurately interpret what is being said (explicitly and implicitly)<br><br>Cluster 5; statement 37. Putting people at ease and building rapport through the effective use of introductions to clarify roles, positive interested body language, gentle use of humour, and other mechanisms
1.5 Manage disagreements and emotionally charged conversations	Cluster 5; statement 4. Skills in negotiation and conflict resolution, including the ability to challenge in a non-confrontational manner and to view conflict as a source of learning and innovation
1.6 Adapt to the unique needs and preferences of each patient and to his or her clinical condition and circumstances	Cluster 6; statement 11. An agile and pragmatic approach to the delivery of individualised care according to the desired goals of the patient. Includes a responsiveness to changing needs of the patient and an understanding that a patient's capacity to make decisions changes over time
2.1 Use patient-centred interviewing skills to effectively gather relevant biomedical and psychosocial information	Cluster 4; statement 42. The effective use of questioning and strategies (e.g. paraphrasing, checking) to elicit all important information and ensure shared understanding with the patient<br><br>Cluster 5; statement 44. The ability to listen and accurately interpret what is being said (explicitly and implicitly)
2.2 Provide a clear structure for and manage the flow of an entire patient encounter	*Clinically specific*
2.3 Seek and synthesize relevant information from other sources, including the patient's family, with the patient's consent	Cluster 6; statement 15. Contextual awareness of patients including the social circumstances and spiritual and philosophical beliefs, which drive patients and influence their behaviour and interactions, leading to a holistic view of health and well-being.

3.1 Share information and explanations that are clear, accurate, and timely, while checking for patient and family understanding	Cluster 4; statement 2. A responsiveness to the communication and health literacy needs of the patient, including adaptability to ensure communication is in a manner that patients will understand
3.2 Disclose harmful patient safety incidents to patients and their families accurately and appropriately	*Clinically specific*
4.1 Facilitate discussions with patients and their families in a way that is respectful, non-judgmental, and culturally safe	Cluster 4; statement 25. Embrace cultural diversity and ensure cultural capacity, including the ability to understand, communicate with, and effectively interact with people across cultures, and knowledge of different cultural practices.
4.2 Assist patients and their families to identify, access, and make use of information and communication technologies to support their care and manage their health	*Clinically specific*
4.3 Use communication skills and strategies that help patients and their families make informed decisions regarding their health<br><br>[A very broad statement that could encompass many statements from the concept map]	Cluster 6; statement 34. Recognising that clinical practice is a partnership with patients, and placing importance on patients' opinion about their own health and their management options<br><br>Cluster 4; statement 2. A responsiveness to the communication and health literacy needs of the patient, including adaptability to ensure communication is in a manner that patients will understand<br><br>Cluster 4; statement 42. The effective use of questioning and strategies (e.g. paraphrasing, checking) to elicit all important information and ensure shared understanding with the patient<br><br>Cluster 4; statement 46. The ability to use interpreter services effectively<br><br>Cluster 6; statement 11. An agile and pragmatic approach to the delivery of individualised care according to the desired goals of the patient. Includes a responsiveness to changing needs of the patient and an understanding that a

	patient's capacity to make decisions changes over time<br><br>Cluster 6; statement 15. Contextual awareness of patients including the social circumstances and spiritual and philosophical beliefs, which drive patients and influence their behaviour and interactions, leading to a holistic view of health and well-being.<br><br>Cluster 6; statement 34. Recognising that clinical practice is a partnership with patients, and placing importance on patients' opinion about their own health and their management options<br><br>Cluster 6; statement 50. Treating health care as a shared journey between the patient and all of those who support and care for them, with a willingness and ability to work with patients in order to co-create health and well-being; with this comes an understanding that healthcare is not only the impartation of
5.1 Document clinical encounters in an accurate, complete, timely, and accessible manner, in compliance with regulatory and legal requirements	*Clinically specific*
5.2 Communicate effectively using a written health record, electronic medical record, or other digital technology	Cluster 5; statement 33. Literacy in computers and modern technologies, with the ability to efficiently use technology to facilitate communication (Moved from Cluster 1 Value led professionalism and leadership)<br><br>Cluster 5; statement 60. Effective written communication skills
5.3 Share information with patients and others in a manner that respects patient privacy and confidentiality and enhances understanding	*Maintaining privacy and confidentiality - Essentially a clinical skill* [Enhances understanding comes under 4.3]

Collaborator

The Collaborator role incorporates statements from the CCM representing clusters 2 (Self-awareness and reflective practice); 4 (Active engagement); 5 (Communication to build and manage relationships); 6 (Patient centredness and advocacy) and 7 (Systems awareness, thinking and contribution).

Statements from CCM have the ability to expand the concepts located within the enabling competencies. For example Collaborator enabling competency 3.1 (Determine when care should be transferred to another physician or health care professional) can be mapped to two statements in the CCM which expands the understanding to require two key attributes/competencies: 1) an appropriate level of both self-confidence and humility with an awareness of limitations (C2/S12), and 2) the knowledge of the broader health care system and skills to assist patients to navigate and utilise all service/expertise available to them. the broader health care team to (C6/S51).

1. Work effectively with physicians and other colleagues in the health care professions<br>2. Work with physicians and other colleagues in the health care professions to promote understanding, manage differences, and resolve conflicts<br>3. Hand over the care of a patient to another health care professional<br>to facilitate continuity of safe patient care

1.1 Establish and maintain positive relationships with physicians and other colleagues in the health care professions to support relationship-centred collaborative care	Cluster 4; statement 57. Ensure, through effective collaboration with colleagues and communication with patients, that there is a seamless transfer of care through the system<br>Cluster 5; statement 10. Clear, purposeful, timely and actively open communication channels with colleagues ensuring a shared understanding of patient's needs between members of the health care team and that concerns can be expressed, including being appropriately available for consultation with colleagues as required
1.2 Negotiate overlapping and shared responsibilities with physicians and other colleagues in the health care professions in episodic and ongoing care	Cluster 7; statement 47. The ability to execute a specific role within a broader system, with recognition of that role as an integral component of a complex system

	Cluster 7; statement 49. The ability to work collaboratively with all clinical colleagues, which includes: an understanding of the role of self and others in the health professional team; a disposition to engage allied health professionals in the care of patients and value the care that is provided by allied health; and taking on a coordinating leadership role where appropriate
1.3 Engage in respectful shared decision-making with physicians and other colleagues in the health care professions	Cluster 5; statement 29. An attitude and approach to relationships with co-workers and patients that is respectful, friendly, non-judgemental, positive and encouraging.<br><br>38. Being able to manage differences in agenda between members of the health team, including the patient
2.1 Show respect toward collaborators	Cluster 5; statement 6. An approach to interpersonal relationships with colleagues that is judicious, empathetic, and responsive to interpersonal dynamics to ensure effective working relationships
2.2 Implement strategies to promote understanding, manage differences, and resolve conflicts in a manner that supports a collaborative culture	Cluster 5; statement 4. Skills in negotiation and conflict resolution, including the ability to challenge in a non-confrontational manner and to view conflict as a source of learning and innovation<br><br>38. Being able to manage differences in agenda between members of the health team, including the patient
3.1 Determine when care should be transferred to another physician or health care professional	Cluster 6; statement 51. Knowledge and ability to assist patients to navigate and utilise the broader health care team to ensure all necessary expertise is available to them<br><br>Cluster 2; statement 12. Appropriate self-confidence, humility and awareness of limitations.
3.2 Demonstrate safe handover of care, using both verbal and written communication, during a patient transition to a different health care professional, setting, or stage of care	*Handover – specific clinical skill [made up of a number of other competencies which are highlighted in the CCM – eg working relationship with colleagues, communication] but primarily a clinical school*

Leader

Enabling competencies can be mapped to CCM statements across clusters 1,2,3 and 7. Leadership in CanMEDS incorporates the CCM elements of role-modelling, self-awareness and reflection – particularly pertaining to patient safety and error and self care, cognitive capability, and systems awareness and thinking. Resource allocation is identified in CanMED but not CCM. The leadership cluster in CCM (cluster 1) also incorporates professional attributes (empath, compassion, integrity, commitment) and an ability and willingness to take initiative. The mapping process highlights that enabling competency 1.2 (contribute to a culture that promotes patient safety) begins with reflective practice on own errors and self-directed ability to learn iteratively throughout career and to use this to improve quality. CCM also highlights the need for advocacy as an important attribute to contribute to system change.

Key competencies

1. Contribute to the improvement of health care delivery in teams, organizations, and systems<br>2. Engage in the stewardship of health care resources<br>3. Demonstrate leadership in professional practice<br>4. Manage career planning, finances, and health human resources in a practice

Enabling competencies

1.1 Apply the science of quality improvement to contribute to improving systems of patient care	Cluster 7; statement 16. An understanding of systems science, leading to a preparedness to tackle the challenges of health systems including ability to analyse health care from a systems perspective and providing feedback about system related concerns
1.2 Contribute to a culture that promotes patient safety	Cluster 2; statement 45. The ability to recognise and critically reflect on own mistakes, embrace failure and adversity as an opportunity for improvement, accept advice and guidance, take criticism seriously but not personally.<br><br>Cluster 3; statement 7. Self-directed ability to identify and act on learning opportunities iteratively and throughout career, including the use of feedback (including from patients), evaluation and audit of practice and patient data as an impetus for quality improvement of practice

1.3 Analyze patient safety incidents to enhance systems of care	*Clinical competency*
1.4 Use health informatics to improve the quality of patient care and optimize patient safety	? *Clinically specific*. Reflected more broadly in competencies outlined in Cluster 2. Attributes for self-awareness and reflective practice – is the clinically relevant aspect of Enabling competency 4.3
2.1 Allocate health care resources for optimal patient care	Not represented
2.2 Apply evidence and management processes to achieve cost-appropriate care	Not represented
3.1 Demonstrate leadership skills to enhance health care<br>[Broad, could include the whole cluster 1]	Cluster 1; statement 23. Effective role-modelling and leadership
3.2 Facilitate change in health care to enhance services and outcomes	Cluster 7; statement 22. Courage to advocate for change or improvement when required even under adverse circumstances (moved from Cluster 4 Active engagement)
4.1 Set priorities and manage time to integrate practice and personal life	Cluster 2; statement 43. A skill set and lifestyle that ensures own well-being and an appropriate work-life balance
4.2 Manage a career and a practice	
4.3 Implement processes to ensure personal practice improvement	Cluster 2; statement 19. Insight into and capacity to reflect on own behaviour and awareness of how it impacts on others<br>Cluster 2; statement 45. The ability to recognise and critically reflect on own mistakes, embrace failure and adversity as an opportunity for improvement, accept advice and guidance, take criticism seriously but not personally.

Enabling competency 4.3 (Leader) Implement processes to ensure personal practice improvement when mapped to the CCM statements it becomes apparent hat this requires insight into and capacity to reflect on own behaviour and mistakes, to embrace adversity as an opportunity for improvement, and accept advice and guidance without taking criticism seriously (Cluster 2, statements 19 and 45).

Health Advocate

The Health Advocate role maps to clusters 2, 3, 6 and 7

Enabling competency 1.2 (Health Advocate) :"Work with patients and their families to increase opportunities to adopt healthy behaviours" when mapped to the CCM requires a contextual awareness of patients' circumstances, believes and values that drive their behaviour (Cluster 6, statement 15), in addition to treating health care as a shared journey which leads to the co-creation of health and well-being – that healthcare is not only the impartation of knowledge (Cluster 6, statement 50).

1. Respond to an individual patient's health needs by advocating with the patient within and beyond the clinical environment<br>2. Respond to the needs of the communities or populations they serve by advocating with them for system-level change in a socially accountable manner

Enabling competencies

1.1 Work with patients to address determinants of health that affect them and their access to needed health services or resources	Cluster 6; statement 51. Knowledge and ability to assist patients to navigate and utilise the broader health care team to ensure all necessary expertise is available to them<br>Cluster 6; statement 3. A willingness and understanding of how to advocate for patients' interests
1.2 Work with patients and their families to increase opportunities to adopt healthy behaviours	Cluster 6; statement 15. Contextual awareness of patients including the social circumstances and spiritual and philosophical beliefs, which drive patients and influence their behaviour and interactions, leading to a holistic view of health and well-being.<br>Cluster 6; statement 50. Treating health care as a shared journey between the patient and all of those who support and care for them, with a willingness and ability to work with patients in order to co-create health and well-being; with this comes an understanding that healthcare is not only the impartation of
1.3 Incorporate disease prevention, health promotion, and health surveillance into interactions with individual patients	*Clinically specific*

2.1 Work with a community or population to identify the determinants of health that affect them	Cluster 7; statement 41. Social awareness and responsibility, with a willingness and skill set to engage with the local community and contribute in ways other than direct clinical work – is only statement that maps to 2.1 and 2.3 – so these are recognised as the same in CCM
2.2 Improve clinical practice by applying a process of continuous quality improvement to disease prevention, health promotion, and health surveillance activities	Cluster 2; statement 45. The ability to recognise and critically reflect on own mistakes, embrace failure and adversity as an opportunity for improvement, accept advice and guidance, take criticism seriously but not personally.<br><br>Cluster 3; statement 7. Self-directed ability to identify and act on learning opportunities iteratively and throughout career, including the use of feedback (including from patients), evaluation and audit of practice and patient data as an impetus for quality improvement of practice
2.3 Contribute to a process to improve health in the community or population they serve	Cluster 7; statement 41. Social awareness and responsibility, with a willingness and skill set to engage with the local community and contribute in ways other than direct clinical work

Scholar

Clusters 1, 3, 4 ,5 ,7.

1.Engage in the continuous enhancement of their professional activities through ongoing learning<br>2. Teach students, residents, the public, and other health care professionals<br>3. Integrate best available evidence into practice<br>4. Contribute to the creation and dissemination of knowledge and practices applicable to health

CCM identifies the attributes that are required to implement enabling competencies. For example enabling competency 1.1 (Scholar) Develop, implement, monitor, and revise a personal learning plan to enhance professional practice maps to three statements across clusters 2(attributes for self awareness and reflective practice) and 3 (Cognitive capability) and highlights the requirement of being curious and observant, having flexibility of thought when faced with new evidence, to incorporate new evidence into practice, and to manage ability to manage uncertainty,

Enabling competencies

1.1 Develop, implement, monitor, and revise a personal learning plan to enhance professional practice	Cluster 2; statement 17. Being curious, observant and notice things<br><br>Cluster 3; statement 28. Flexibility of thought when faced with new evidence and incorporate innovations into practice<br><br>Cluster 3; statement 5. Ability to manage uncertainty and ambiguity: the ability to learn, reflect and develop in the face of situations where there is no known, reliable or definitive answer; or that are complex or unfamiliar.
1.2 Identify opportunities for learning and improvement by regularly reflecting on and assessing their performance using various internal and external data sources	Cluster 3; statement 5. Ability to manage uncertainty and ambiguity: the ability to learn, reflect and develop in the face of situations where there is no known, reliable or definitive answer; or that are complex or unfamiliar.
1.3 Engage in collaborative learning to continuously improve personal practice and contribute to collective improvements in practice	Cluster 7; statement 49. The ability to work collaboratively with all clinical colleagues, which includes: an understanding of the role of self and others in the health professional team; a disposition to engage allied health professionals in the care of patients and value the care that is provided by allied health; and taking on a coordinating leadership role where appropriate

2.1 Recognize the influence of role-modelling and the impact of the formal, informal, and hidden curriculum on learners	Cluster 1; statement 23. Effective role-modelling and leadership<br><br>Cluster 7; statement 21. Contribute to the education of others through actions in day-to-day activities
2.2 Promote a safe learning environment	Cluster 5; statement 6. An approach to interpersonal relationships with colleagues that is judicious, empathetic, and responsive to interpersonal dynamics to ensure effective working relationships
2.3 Ensure patient safety is maintained when learners are involved	*Clinically specific*
2.4 Plan and deliver a learning activity	*Clinically specific*
2.5 Provide feedback to enhance learning and performance	Cluster 5; statement 36. Provide feedback to others in an effective non-confrontational manner when required
2.6 Assess and evaluate learners, teachers, and programs in an educationally appropriate manner	*Clinically specific*
3.1 Recognize practice uncertainty and knowledge gaps in clinical and other professional encounters and generate focused questions that address them	Cluster 3; statement 5. Ability to manage uncertainty and ambiguity: the ability to learn, reflect and develop in the face of situations where there is no known, reliable or definitive answer; or that are complex or unfamiliar.<br><br>*Research competency*
3.2 Identify, select, and navigate pre-appraised resources	Cluster 3; statement 40. Resourcefulness: ability to identify and access information and resources to aid clinical care.
3.3 Critically evaluate the integrity, reliability, and applicability of health-related research and literature	*Clinically specific*
3.4 Integrate evidence into decision-making in their practice	Cluster 3; statement 55. Ability for decisive action by assessing relevant information, putting this into perspective of other considerations, weighing up the risk and benefit and acting accordingly (Moved from Cluster 2 – Attributes for self-awareness and reflective practice)
4.1 Demonstrate an understanding of the scientific principles of research and scholarly inquiry and the role of research evidence in health care	*Clinically specific*

4.2 Identify ethical principles for research and incorporate them into obtaining informed consent, considering potential harms and benefits, and considering vulnerable populations	*Clinically specific*
4.3 Contribute to the work of a research program	*Clinically specific*
4.4 Pose questions amenable to scholarly inquiry and select appropriate methods to address them	*Clinically specific*
4.5 Summarize and communicate to professional and lay audiences, including patients and their families, the findings of relevant research and scholarly inquiry	*Clinically specific*

Professional

The Professional role in CanMEDS is mapped to cluster 1 and 2 of the CCM, meaning that elements of professionalism and leadership, self-awareness and reflective practice, and communication to build and manage relationships underlie the CanMEDS Professional role. CCM cluster analysis brings together the two CanMED roles of professional and leader – indicating that they are seen by our participants as intersecting but separate roles. Self-awareness of own motivations is highlighted by CCM as an important aspect of managing conflicts of interest; self-awareness also highlighted in ensuring own well-being. Professionalism in CCM ….

Clusters

1.Demonstrate a commitment to patients by applying best practices and adhering to high ethical standards<br>2. Demonstrate a commitment to society by recognizing and responding to societal expectations in health care<br>3. Demonstrate a commitment to the profession by adhering to standards and participating in physician-led regulation<br>4. Demonstrate a commitment to physician health and well-being to foster optimal patient care

Enabling competencies

1.1 Exhibit appropriate professional behaviours and relationships in all aspects of practice, demonstrating honesty, integrity, humility, commitment, compassion, respect, altruism, respect for diversity, and maintenance of confidentiality	Cluster 1; statement 1. A professional commitment with strong work ethic, self-motivation, and an intention to make a difference for patients and the community<br>Cluster 1; statement 31. Integrity – honest, strong moral principles and trustworthy
1.2 Demonstrate a commitment to excellence in all aspects of practice	Cluster 1; statement 1. A professional commitment with strong work ethic, self-motivation, and an intention to make a difference for patients and the community
1.3 Recognize and respond to ethical issues encountered in practice	Not identified overtly
1.4 Recognize and manage conflicts of interest	Cluster 2; statement 13. Self-awareness and understanding of own motivations, responses, biases and emotional triggers. Ensuring that these things don't impact on patient care and that actions are always in the best interest of patients

1.5 Exhibit professional behaviours in the use of technology-enabled communication	~~Cluster 5; statement 33. Literacy in computers and modern technologies, with the ability to efficiently use technology to facilitate communication (Moved from Cluster 1 Value led professionalism and leadership)~~<br>[Not what they're getting at. Relates to use of social media which is not explicitly covered.
2.1 Demonstrate accountability to patients, society, and the profession by responding to societal expectations of physicians	Cluster 1; statement 35. Professional conduct in a manner that is consistent with the community's expectations: A set of values, behaviours, and relationships that underpin trust from the public
2.2 Demonstrate a commitment to patient safety and quality improvement	*Clinically specific*
3.1 Fulfill and adhere to the professional and ethical codes, standards of practice, and laws governing practice	Ethical behaviour presents in our model implicitly in attributes, but not explicitly as here
3.2 Recognize and respond to unprofessional and unethical behaviours in physicians and other colleagues in the health care professions	Cluster 5; statement 36. Provide feedback to others in an effective non-confrontational manner when required
3.3 Participate in peer assessment and standard-setting	*Clinically specific*
4.1 Exhibit self-awareness and manage influences on personal well-being and professional performance	Cluster 2; statement 43. A skill set and lifestyle that ensures own well-being and an appropriate work-life balance
4.2 Manage personal and professional demands for a sustainable practice throughout the physician life cycle	Cluster 1; statement 53. Willingness and ability to take initiative; including handling responsibilities and duties without waiting for instruction as appropriate, and taking responsibility for personal growth and development
4.3 Promote a culture that recognizes, supports, and responds effectively to colleagues in need	Cluster 1, statement 23. . Effective role-modelling and leadership<br>Cluster 1, statement 26. Empathy and compassion – the ability to understand and share the feelings of others, have sympathy for another's misfortune and a desire to alleviate suffering

3. Clusters 1-7 from the Concept Map

Cluster 1. Value led professionalism and leadership

Statements in cluster:

1. A professional commitment with strong work ethic, self-motivation, and an intention to make a difference for patients and the community
23. Effective role-modelling and leadership
24. Efficiency of work practices and good organisational skills
26. Empathy and compassion - the ability to understand and share the feelings of others, have sympathy for another's misfortune and a desire to alleviate suffering
31. Integrity - honest, strong moral principles and trustworthy
35. Professional conduct in a manner that is consistent with the community's expectations: A set of values, behaviours, and relationships that underpin trust from the public
53. Willingness and ability to take initiative; including handling responsibilities and duties without waiting for instruction as appropriate, and taking responsibility for personal growth and development [Not well covered in CanMEDS]
54. Willingness to make time when time is needed

Cluster 2. Attributes for self-awareness and reflective practice

Statements in cluster:

12. Appropriate self-confidence, humility and awareness of limitations.

13. Self-awareness and understanding of own motivations, responses, biases and emotional triggers. Ensuring that these things don't impact on patient care and that actions are always in the best interest of patients
17. Being curious, observant and notice things
19. Insight into and capacity to reflect on own behaviour and awareness of how it impacts on others
39. Resilience - the ability to recover from adversity
43. A skill set and lifestyle that ensures own well-being and an appropriate work-life balance
45. The ability to recognise and critically reflect on own mistakes, embrace failure and adversity as an opportunity for improvement, accept advice and guidance, take criticism seriously but not personally.
59. Perseverance and patience.

Cluster 3. Cognitive capability

Statements in cluster:

5. Ability to manage uncertainty and ambiguity: the ability to learn, reflect and develop in the face of situations where there is no known, reliable or definitive answer; or that are complex or unfamiliar.
7. Self-directed ability to identify and act on learning opportunities iteratively and throughout career, including the use of feedback (including from patients), evaluation and audit of practice and patient data as an impetus for quality improvement of practice
9. Flexibility and adaptability to adapt to diverse, challenging and changing environments
18. Creative and innovative approach to solving complex problems. Being open minded to possibilities when problem solving, be prepared to consider different points of view and to look for different solutions when required.
20. Metacognition: Higher order clarity of thought processes with control over cognitive processes through understanding and analysis. (Moved from Cluster 2 – Attributes for self-awareness and reflective practice)
28. Flexibility of thought when faced with new evidence and incorporate innovations into practice
30. Situational awareness: insightful with respect to the environment in space and time.
40. Resourcefulness: ability to identify and access information and resources to aid clinical care.
55. Ability for decisive action by assessing relevant information, putting this into perspective of other considerations, weighing up the risk and benefit and acting accordingly (Moved from Cluster 2 – Attributes for self-awareness and reflective practice)
56. Clarity of thought and ability to remain calm and take appropriate actions when under pressure

Cluster 4. Active engagement

Statements in cluster:

2. A responsiveness to the communication and health literacy needs of the patient, including adaptability to ensure communication is in a manner that patients will understand
25. Embrace cultural diversity and ensure cultural capacity, including the ability to understand, communicate with, and effectively interact with people across cultures, and knowledge of different cultural practices.
42. The effective use of questioning and strategies (e.g. paraphrasing, checking) to elicit all important information and ensure shared understanding with the patient
46. The ability to use interpreter services effectively [Not explicit in CanMEDS]
48. Understand the importance of narrative in medicine; to engage in narrative with patients and use these narratives to inform the care provided
57. Ensure, through effective collaboration with colleagues and communication with patients, that there is a seamless transfer of care through the system

Cluster 5. Communication to build and manage relationships

Statements in cluster:

4. Skills in negotiation and conflict resolution, including the ability to challenge in a non-confrontational manner and to view conflict as a source of learning and innovation
6. An approach to interpersonal relationships with colleagues that is judicious, empathetic, and responsive to interpersonal dynamics to ensure effective working relationships
10. Clear, purposeful, timely and actively open communication channels with colleagues ensuring a shared understanding of patient's needs between members of the health care team and that concerns can be expressed, including being appropriately available for consultation with colleagues as required
27. Ability to engender trust from colleagues and patients
29. An attitude and approach to relationships with co-workers and patients that is respectful, friendly, non-judgemental, positive and encouraging.
33. Literacy in computers and modern technologies, with the ability to efficiently use technology to facilitate communication (Moved from Cluster 1 Value led professionalism and leadership)
36. Provide feedback to others in an effective non-confrontational manner when required
37. Putting people at ease and building rapport through the effective use of introductions to clarify roles, positive interested body language, gentle use of humour, and other mechanisms
38. Being able to manage differences in agenda between members of the health team, including the patient
44. The ability to listen and accurately interpret what is being said (explicitly and implicitly)
60. Effective written communication skills

Cluster6. Patient-centredness and advocacy

Statements in cluster:
3. A willingness and understanding of how to advocate for patients' interests
8. Ability to establish and maintain mutually respectful relationships with patients valuing personhood
11. An agile and pragmatic approach to the delivery of individualised care according to the desired goals of the patient. Includes a responsiveness to changing needs of the patient and an understanding that a patient's capacity to make decisions changes over time
15. Contextual awareness of patients including the social circumstances and spiritual and philosophical beliefs, which drive patients and influence their behaviour and interactions, leading to a holistic view of health and well-being.
34. Recognising that clinical practice is a partnership with patients, and placing importance on patients' opinion about their own health and their management options
50. Treating health care as a shared journey between the patient and all of those who support and care for them, with a willingness and ability to work with patients in order to co-create health and well-being; with this comes an understanding that healthcare is not only the impartation of knowledge.
51. Knowledge and ability to assist patients to navigate and utilise the broader health care team to ensure all necessary expertise is available to them
52. Willingness and ability to prioritise activities for the benefit of patients

Cluster 7. Systems awareness, thinking and contribution

Statements in cluster:

14. Awareness of the organisational aspects of health care systems, including own responsibilities and the decision-making structures within the system
16. An understanding of systems science, leading to a preparedness to tackle the challenges of health systems including ability to analyse health care from a systems perspective and providing feedback about system related concerns
21. Contribute to the education of others through actions in day-to-day activities
22. Courage to advocate for change or improvement when required even under adverse circumstances (moved from Cluster 4 Active engagement)
32. Knowledge of the broader social systems and services within which health care operates, enabling interaction with them for the benefit of patient care.
41. Social awareness and responsibility, with a willingness and skill set to engage with the local community and contribute in ways other than direct clinical work
47. The ability to execute a specific role within a broader system, with recognition of that role as an integral component of a complex system
49. The ability to work collaboratively with all clinical colleagues, which includes: an understanding of the role of self and others in the health professional team; a disposition to engage allied health professionals in the care of patients and value the care that is provided by allied health; and taking on a coordinating leadership role where appropriate
58. Commitment to work as part of a team to meet legislative and accreditation requirements
